# Benefits of applying X-ray computed tomography in bentonite based material research focussed on geological disposal of radioactive waste

**DOI:** 10.1007/s11356-020-08151-2

**Published:** 2020-03-02

**Authors:** Heini Maria Reijonen, Jukka Kuva, Pasi Heikkilä

**Affiliations:** Geological Survey of Finland (GTK), PL 96, FI-02151 Espoo, Finland

**Keywords:** X-ray computed tomography (XCT), Bentonite, Geological disposal, Radioactive waste, Natural analogue, Geomaterial

## Abstract

Bentonite-based geomaterials are included in the designs of geological repository planning in most countries, especially in high-level radioactive waste disposal. Physical integrity of the bentonite sealant is key in assuring its hydraulic and retention properties, which affect the long-term performance of the repositories. Examination of the internal textures and structures of bentonite has been challenging until recently. Here, X-ray computed tomography (XCT) is applied to improve the textural and structural characterization of natural and man-made bentonite samples. Based on these initial analyses, clear benefits have been identified compared with conventional bentonite research methods. First, applying XCT prior to destructive analytical methods provides means to distinguish secondary features or in situ textures. It allows to eliminate false interpretations due to sample deformation and guides subsampling. Second, XCT images add the third dimension to analyses, allowing larger spatial coverage in less time. Overall, findings support the application of XCT for reducing uncertainties related to physical characterization of bentonite samples, both natural and industrial. They also show that XCT has potential to be developed to support quality assurance processes for bentonite sealant manufacturing.

**Electronic supplementary material:**

The online version of this article (10.1007/s11356-020-08151-2) contains supplementary material, which is available to authorized users.

## Introduction

Utilizing clay materials in the geotechnical applications for geological disposal of high level radioactive waste has one major difference to all other environmental applications—the time frame of assessing the performance of the systems spans up to a million years. This sets a requirement to understand processes that are beyond experimental timescales. The only way to study processes in geological timescales is through field investigations on the deposits themselves. The most common clay type planned to be used in these geotechnical solutions is bentonite in various forms. Depending on the repository design, bentonite can be used as dried and ground, or further modified (e.g. via cation exchange). In addition, in some designs, mixtures of bentonite, sand and/or aggregates are considered. In order to assess long-term performance of these materials, geological formations of bentonite have been studied in the past (often referred to as natural analogues, NA, see e.g. Reijonen and Alexander 2015), but no detailed large-scale textural analyses have been produced to date. Here, the X-ray computed tomography (XCT) method is applied to bentonites, providing a new, structurally more profound way to interpret the results of physical and chemical analyses of samples. The study is a compilation of three phases: 1) initial assessment of the method supported by experimental data (Alexander et al. 2017), 2) applying the method in the initial phase for new NA investigations and 3) applying the method on engineered samples to enable comparison.

## Background

To date, XCT has been applied in bentonite material investigations related to water uptake and deformation (e.g. Harjupatana et al. 2015; Molinero Guerra 2018), desiccation and cracking (e.g. Gebrenegus et al. 2011), microstructure description and swelling behaviour of bentonite-sand mixtures (e.g. Saba et al. 2014; Sarkar and Sumi 2016) and purified processed bentonite characterization (e.g. Kozaki 2001). The method has also been employed in experiments investigating other swelling clays, such as beidellite-kaolinite pellet hydration (Van Geet et al. 2005). Kawaragi et al. (2009) used XCT to characterize both processed and natural bentonite samples, but for the use in large scale data acquisition for deposit-wide bentonite petrography, the method is yet to be employed (although other types of clay deposits have previously been studied, e.g. Boom Clay: Hemes 2015).

One aspect of particular relevance for XCT and its use in bentonite characterization is to consider sampling artefacts. Sampling of bentonite can cause deformation during extraction from a deposit through heave when confinement is lost or by deformation during drilling due to mechanical strain by the drill bit, even if drilled without water. Drilling itself also deforms soft material especially at the edges of the samples, as observed by Alexander et al. (2017). Further deformation may be caused if samples are transported or stored unprotected against humidity changes or by impact damage (e.g. from dropping). Even further deformation can be caused by the impregnation methods used in thin section manufacturing (all observed by Alexander et al. 2017). These artefacts can often be easily observed in the samples and the damage can be taken into account in the interpretation, but less disturbed samples would be preferred (see discussion in Ewy 2015). In this study, the emphasis is set on identifying these artefacts via XCT and assessing their significance to the interpretation of the results (those presented in Alexander et al. 2017).

**Fig. 1:**
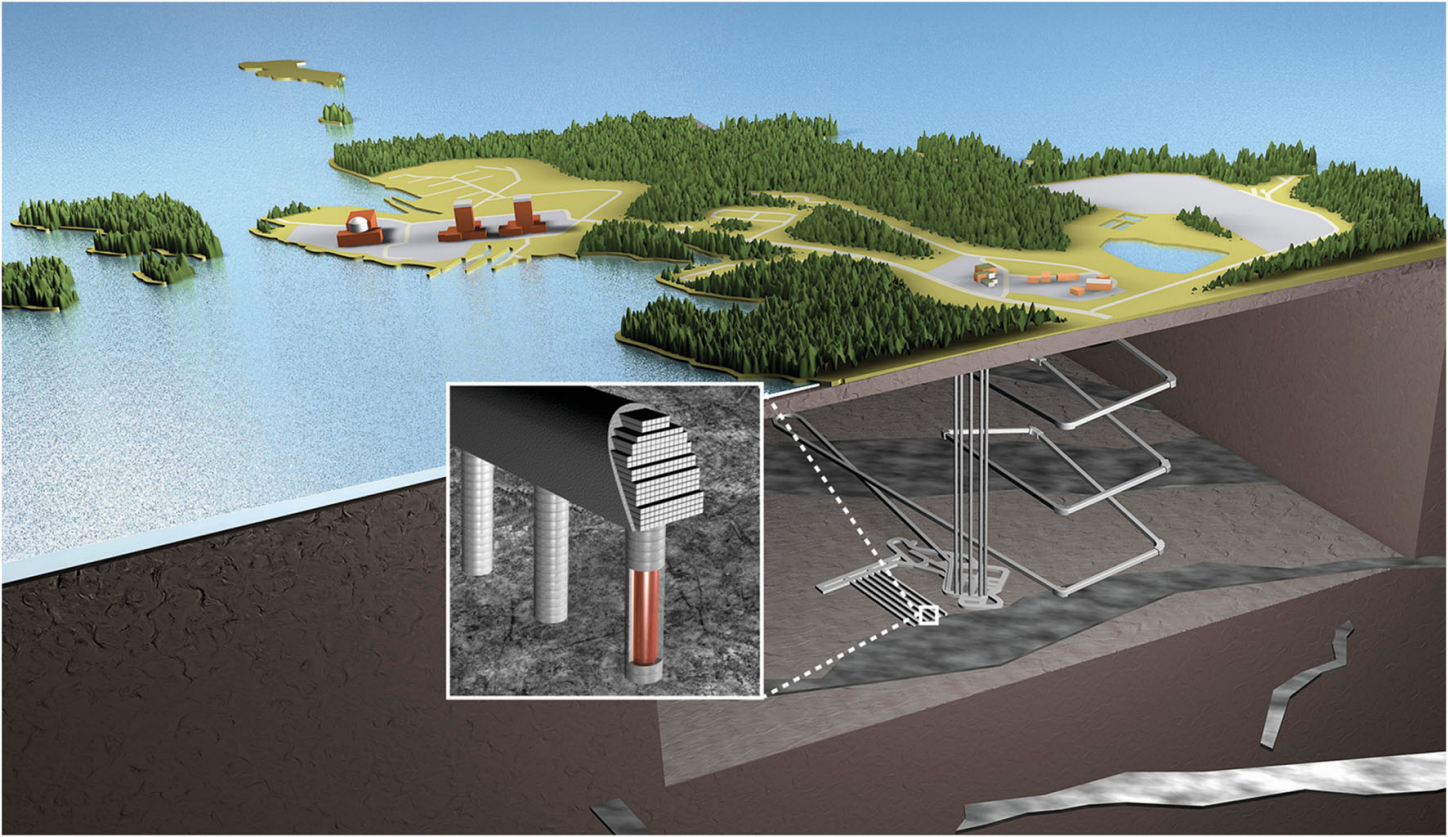
An example of a geological repository design that utilizes processed bentonite in the Engineered Barrier System (EBS). Bentonite is used as sealant around the waste packages (1) and in the tunnel backfills (2), (image courtesy of Posiva Oy)

Bentonites have been studied as NAs for the engineered bentonite materials for decades (see e.g. Reijonen and Alexander 2015 for a review). In the context of NAs, the focus of the research is in understanding the long-term processes involved in the evolution of natural bentonite deposits. They provide an analogy to the processes that could occur in the geological repository for radioactive waste, where processed bentonite is used as backfill/sealant around the waste packages and to backfill tunnels (Fig. 1). Selection of bentonite as a sealing material is based on its good isolation and containments properties (e.g. plasticity, swelling capacity, low hydraulic conductivity, retardation of many chemical substances). These favourable properties are based on the mineralogical composition, density and homogeneity of bentonite. In general, for geological repositories, processed bentonite can be in various forms, for example as bentonite granules (crushed bentonite), pre-compacted blocks or pellets (e.g. Sinnathamby et al. 2015). In addition, bentonite mixtures (with sand or other aggregates) are being considered (e.g. Chen et al. 2017).

Bentonites are formed by hydrothermal or diagenetic alteration of volcanic ash, or other, often volcanogenic rocks (for an overview, see Christidis and Huff 2009). While most commercially produced bentonites are quarried from surficial deposits (e.g. the Kato Moni site discussed here), some rare underground mines also exist. For example, the Tsukinuno deposit in Japan, discussed here, reaches a depth of several hundred metres. The usability of NAs in the future performance assessments of repository systems (Fig. 1) rely on the overall understanding of the similarities of the systems, including physical and chemical similarities of the bentonite itself and the surrounding environment.^1^ This aspect has not been the prime focus in most bentonite NA investigations. Here, XCT analysis is tested for its potential to increase deposit scale understanding of the natural bentonites and to provide a non-destructive tool to compare natural and processed bentonites.

## Samples

In this study, samples from natural bentonite deposits from the Kato Moni quarry in Cyprus, the Tsukinuno mine in Japan, and Dobuyama quarry in Japan, along with two samples of processed bentonites, were investigated (Table 1). All samples were stored and analysed at room temperature.

**Table 1:** Samples investigated in this study by means of XCT. Mineralogy abbreviations: smectite (S), mica (M), kaolinite (K), laumonite (L), quartz (Q), plagioclase (P), ankerite (A), calcite (C), pyrite (PY), small letters denote occurrence as scarce/uncertain

Sample ID	Location/product name	Type/mineralogy	Sample size	Notes	Dry density g cm^−3^	Initial water content %	Bulk density g cm^−3^	Reference for mineralogy/density/water content
KM1-B4a (9.70–9.76 m)	Kato Moni quarry, Cyprus	Natural bentonite (S + Q + P + C + k + m + py)	Drill core quarter	Samples have been stored in sealed plastic packages and in plastic box after sampling in 2015. Unconfined samples, swelling after sampling due to pressure release. Sampling depth indicated in Sample ID.	1.51	22.4*	1.85	Alexander et al. 2017 Values elected from Appendix D for corresponding depth range as the original sampling depth.
KM1-B4b		Natural bentonite (S + Q + P + C + k + m + py)	Sub-sample (small Ø < 5 mm)		as above	as above		
KM1-B7 (11.61–11.66 m)		Natural bentonite (S + Q + P + C + m + k + l)	Drill core sector		1.46	29*	1.88	
KM2-B4 (14.65–14.80 m)		Natural bentonite (S + Q + P + C + m + k + py)	Drill core sector		1.55	23.5*	1.91	
KM3-B1 (0.25–0.35 m)		Natural bentonite (C + S + Q + m + k + l + a)	Drill core quarter		1.09**	21.2–22.4*	1.34	
KM3-B5 (2.11–2.19 m)		Natural bentonite (S + Q + P + m + k + l)	Drill core half		1.30**	30.7*	1.70	
T1 (bed #29, depth from surface ~ 220 m)	Tsukinuno mine, Japan	Natural sandy bentonite	cut sub-sample (Ø < 2 cm)	Sampled directly from the mine	1.90	12.85	2.20	Average values for individual bentonite beds. Unpublished Kunimine Industries Company (KIC) data (Ito, pers. comm.)
TM1 (bed #19, depth from surface ~ 230 m)		Natural massive bentonite	cut sub-sample (Ø < 2 cm)	Sampled from mined bentonite heap	1.88	14.24	2.19	
TW1 (bed #31, depth from surface ~ 250 m)		Natural waxy bentonite	cut sub-sample (Ø < 2 cm)	Sampled from mined bentonite heap	1.84	13.38	2.21	
D1	Dobuyama quarry, Japan	Natural pelletal bentonite	hand specimen	Samples from quarry face	n/a			
Block1 (04-05B)	MX-80	Prefabricated bentonite block (Na-bentonite)	Cored sampleØ ~ 5 cm length ~ 12 cm	Sample from prefabricated bentonite blocks with partial saturation	1.81 ± 0.04			na
Pellet1	Bentonite	Prefabricated bentonite pellet sample (detailed mineralogy not available)	Ø ~ 5–10 mm	Commercial bentonite pellets	n/a			na

*Some drying of samples may have occurred during storage before XCT measurement (stored in plastic wrapping)

**KM3 drill hole has been drilled directly in exposed bentonite at the base of the quarry and it is possible that it has been disturbed by the quarrying activities

### Phase I samples

Alexander et al. (2017) provided a detailed textural description of the Cypriot Kato Moni bentonite, using optical petrography and backscattered scanning electron microscopy (BSEM) to examine epoxy resin impregnated thin sections with blue dye (small 48 × 28 mm and large format 78 × 50 mm). Their study has been used as a basis in assessing the applicability of the XCT method by analysing additional samples collected during the field investigations at Kato Moni.

The Kato Moni bentonite is low grade (smectite content 20.6 to 36.2%) bentonite with calcium as the main exchangeable cation (Alexander et al. 2017). It is part of the youngest sequence of the Troodos ophiolite, deposited on top of the pillow lavas (for a description of the full geological setting, see Alexander et al. 2017).

### Phase II samples

New samples were collected from two bentonite sites in Japan: the Tsukinuno bentonite mine and the Dobuyama quarry. Both sites have been previously investigated and the mineralogical composition is well known (e.g. Itoh et al. 1999; Takagi et al. 2005), but no textural 3D characterization has been undertaken.

The Tsukinuno bentonite is diagenetic miocene high-grade Na-bentonite, although Ca-bentonites occur as well. The deposit in Japan is comprised of 35 bentonite beds with variable properties (e.g. Itoh et al. 1999; Takagi et al. 2005) that occur as layers of variable thickness in hard shale. The Dobuyama bentonite is hydrothermal and possesses clear lapilli tuff type textures in hand specimen. Here, XCT was employed to characterize bentonite samples which, at the hand specimen scale, had been noted to possess varied textures. The mineral composition was also determined by qualitative XRD.

### Phase III samples

**Fig. 2:**
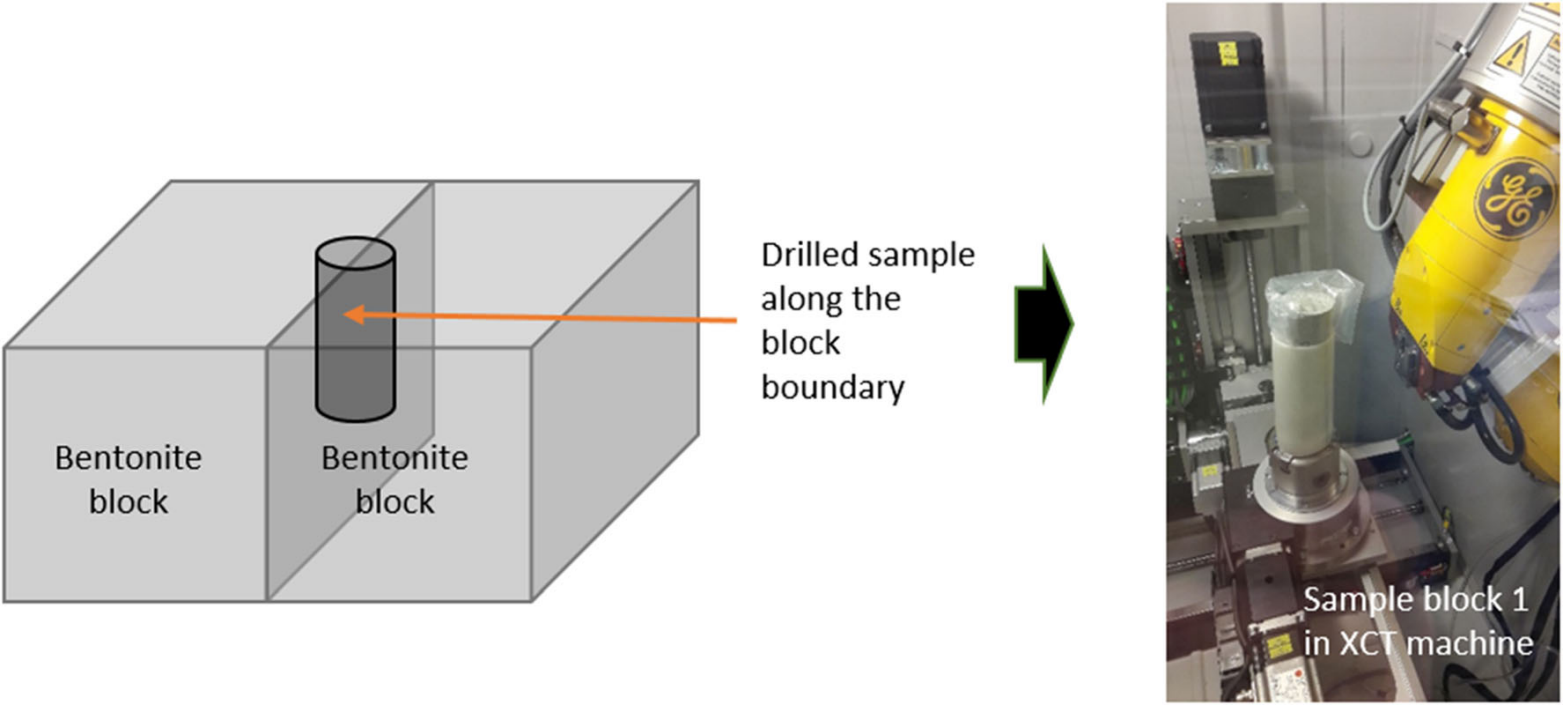
A schematic illustration of the block sampling, sampled by drilling between two bentonite blocks (left) and image of sample still in vacuum in the XCT device (right)

Samples tested here come from a large scale test (Fig. 2) where prefabricated blocks have been stacked and partially saturated (Marjavaara, pers. comm.). The material used in the test has been described in detail by Kiviranta et al. (2018, see sample Be-Wy--BT0020-Sa-R). It is high quality Na-bentonite, with high cation exchange capacity (0.93 eq/kg). Here, XCT is used as a non-destructive method to further characterize samples before full post-mortem analysis, as well as to compare the internal texture of compressed bentonite to natural samples. For this sample, various image resolutions were investigated. Prefabricated blocks of MX-80 bentonite from Wyoming, USA have been used in many experiments to investigate bentonite performance of the repository EBS. In addition, bentonite pellets of different degrees of wetting were included.

## Methods

### XCT

The result of a XCT scan is a cubic 3D lattice of X-ray attenuation coefficients of the sample material. In practice, this is a 3D image, where the grayscale values of voxels (3D pixels) are related to the attenuation coefficients, which are mostly dependent on density, but also on the beam energy and the effective atomic number of the material (Murty 1965). This means that different beam energies can and will produce a resultant image with different contrast between the sample components. This can be crucial when the sample contains components that are close to each other in density, as can be the case in natural bentonite, for example. Therefore, it is important to choose the scan power settings correctly to maximize contrast between different components. Since the X-rays in a laboratory XCT device are polychromatic, there is no straightforward way to accurately deduce absolute densities from the resulting image, but rather relative densities are obtained. Any lighter voxels in the final image indicate higher X-ray absorption and thus a higher density than darker voxels.

All XCT work was conducted using a GE phoenix v|tome|x s, using a 240 kV microfocus tube for all samples except KM1-B4b, which was imaged with a 180 kV nanofocus tube with focus mode 2. The settings utilized for all sample scans except pellet 1 are shown in Table 2, the pellet wetting experimental setup of pellet 1 is described in the next section. Block sample was scanned using 3 different resolutions, labelled in Table 2 and block 1a, 1b and 1c. One sample, block 1 (see Table 1), was scanned while still inside a plastic vacuum bag, to preserve the moisture within the sample and avoid any debris from the sample within the device. This was quite easy, as plastic absorbs X-rays very weakly and the used device was quite spacious.

**Table 2:** XCT settings for all samples apart from pellet1. No beam filter was used for any of the scans. Accelerating voltage, tube current and tube power (product of the previous two) are standard power settings for X-ray tubes

Sample	Accelerating voltage/kV	Tube current/μA	Tube power/W	Total scan time/min	Resolution/μm
KM1-B4a	150	150	22.5	56	25.07
KM1-B4b	60	410	24.6	667	1.44
KM1-B7	100	314	31.4	53	31.43
KM2-B4	100	310	31	60	37.74
KM3-B1	100	310	31	60	33.11
KM3-B5	150	280	42	56	52.79
T1	200	46	9.2	90	9.38
TM1	200	56	11.2	90	11.36
TW1	200	50	10	90	10.10
D1	125	500	62.5	33	50.77
Block 1a	125	510	63.75	13	64.15
Block 1b	125	240	30	45	30.18
Block 1c	100	100	10	180	10.38

### Pellet wetting experimental set up

The pellet experiment was carried out in a glass decanter, first imaging dry bentonite pellets, followed by periodical addition of MilliQ water. After wetting, the container was closed with a paraffin film. The sample was measured at several time steps (Table 3) during the experiment in a 3D and in a 4D (3D + time) experiment using the normal scan mode and the fast|scan feature of the XCT device, where the sample rotates constantly and images are taken during rotation. The final scan was a region of interest (ROI) close-up scan (higher resolution). Parameters for different scan types are listed in Table 4. The main difference to the pellet wetting experiment previously reported by Molinero Guerra (2018) was the fact that water was added stepwise, not in a continuous flow. In addition, this experiment was conducted in a volumetrically unrestricted vessel, allowing upward expansion of the pellet mass.

**Table 3:** Time steps and mass of water added to the pellet column

	Action	Scan type	Date/time	Material added	Mass (g)
Step 1	Dry pellets in decanter		0	Pellets	99.29
Step 2	Dry scan	Normal and fast	0		
Step 3	1st wetting		7.5.2018	MilliQ water	61.48
Step 4	Fast scan of saturation	Fast	2, 16, 32, 47, 62, 77 and 92 min after wetting		
Step 5	Normal scan	Normal	106 min after wetting		
Step 6	Normal scan	Normal	After 47 h		
Step 7	2nd wetting		9.5.2018 (2 days)	MilliQ water	78.19
Step 8	Normal scan	Normal	After 7 days		
Step 9	3rd wetting		14.5.2018 (7 days)	MilliQ water	92.8
Step 10	Normal scan	Normal	After 60 days		
Step 11	Region of interest (ROI)	Close-up	After 63 days

**Table 4:** Scan parameters for pellet1. No beam filter was used

Scan type	Fast|scan feature	Accelerating voltage/kV	Tube current/μA	Tube power/W	Total scan time/min	Resolution/μm
Normal	No	150	266	39.9	36	40.04
Fast	Yes	150	266	39.9	1	40.04
ROI	No	150	80	12	90	12.40

### XRD and XRF

The bulk bentonite rock samples for X-ray powder diffraction (XRPD) measurements were taken by selecting representative fragments from homogeneous parts of the solid clay hand samples. The samples were ground in agate mortar in acetone suspension, spread on glass slides and dried. They were measured at GTK (Geological Survey of Finland, Espoo), using Bruker D8 Discover Bragg-Brentano powder diffractometer equipped with Cu-tube, 0.4° fixed divergence slit, 2.5° sollers, beam knife, spinner, Ni-filter and Lynxeye silicon strip detector. Powder diffractograms were measured for 5–70° 2θ using 40 kV and 40 mA power settings in continuous mode, 0.02° 2θ/s and 0.5 s/step. The mineral phases were identified using Bruker EVA software and ICDD (International Centre for Diffraction Data) PDF-4 Minerals (Powder Diffraction File) database. For clay phase identification, each sample was mixed in water and the suspension was allowed to settle overnight. In two test tubes, 1 ml of the surface suspension was mixed with 1 ml 1 M KCl solution and 1 ml 1 M MgCl2 solution that were allowed to react overnight. Excess salt was washed away using centrifuge and ion-exchanged water for four times, verified to be chloride free using AgNO3 chloride test. The solution was pipetted on glass slides and they were allowed to dry slowly to maximize the orientating of the suspended clay flakes. The Mg-ion-exchanged preparation was measured, treated with glycerol and re-measured. The K-ion exchanged preparation was first measured once, then after heating for 1 h in 200 °C and finally after heating for 1 h in 550 °C. The XRPD measurements were done as before, but for 2–35° 2θ in continuous mode, 0.1° 2θ/s and 0.5 s/step.

Semi-quantitative energy dispersive X-ray fluorescence (ED-XRF) analysis was utilized in XRPD phase interpretations. The measurements were done with PANalytical Epsilon 3^XLE^ benchtop instrument equipped with He-flow system for light element analysis. Measurements from small mineral particles were done in plastic sample cups, through 4 μm polypropylene film, utilizing the PANalytical Omnian method that involves 6 measurement channels for each sample. Automated and user-re-interpreted qualitative analysis is followed by quantitative analysis, powered by comprehensive set of correction parameters, including background, line-overlap and fundamental parameter (FP) matrix corrections. It is not possible to provide quantitative results, because of the irregular particle size and distribution and light element signal absorption to the film. Instead, relative compositional differences were complementary to the phase identification by XRPD.

## Results

### Phase I: Kato Moni samples

3D XCT images were produced from all samples analysed. Figure 4 shows a snapshot of a 3D XCT image from the sample KM1-B4a, and Fig. 5 presents the smaller scale sample KM1-B4b (for resolutions, see Table 2). The XCT models were then compared with the petrographic data available from Alexander et al. (2017, Appendix 6). Samples used for XCT were taken adjacent to samples used by Alexander et al. (2017), thus preventing a direct comparison of the observed textures. The XCT samples are, however, taken directly below the thin sections (Table 5), allowing comparison of features visible to the different analytical techniques, such as detection of microfossils, general textural characteristics, intactness of the samples and deformation (primary or secondary).

**Table 5:** Sampling depths of the material studied here and thin section types and sampling depths in Alexander et al. (2017)

Sample ID	Thin section format*	Sampling depth (m.b.g.s.**) (for thin section samples)*	Sampling depth (m.b.g.s.**) (for XCT samples)
KM1-B4	Large (78 × 50 mm)	9.60–9.70 m	9.70–9.80 m
KM1-B7	Normal (48 × 28 mm)	11.55–11.61 m	11.61–11.66 m
KM2-B4	Large (78 × 50 mm)	14.50–14.65 m	14.65–14.80 m
KM3-B1	Normal (48 × 28 mm)	0.17–0.24 m	0.25–0.35 m
KM3-B5	Normal (48 × 28 mm)	2.00–2.10 m	2.11–2.19 m

*Alexander et al. 2017, **Metres below ground surface

The new data obtained from the Kato Moni samples include hand specimens (all samples analysed) and small scale, high resolution analysis (KM1-B4b) in 3D. Both sets of data were used to investigate the benefits and potential problems in the XCT analysis. Here, the main observations are presented with illustrations. Petrographic analysis is fully documented in Online resource 1.

**Fig. 3:**
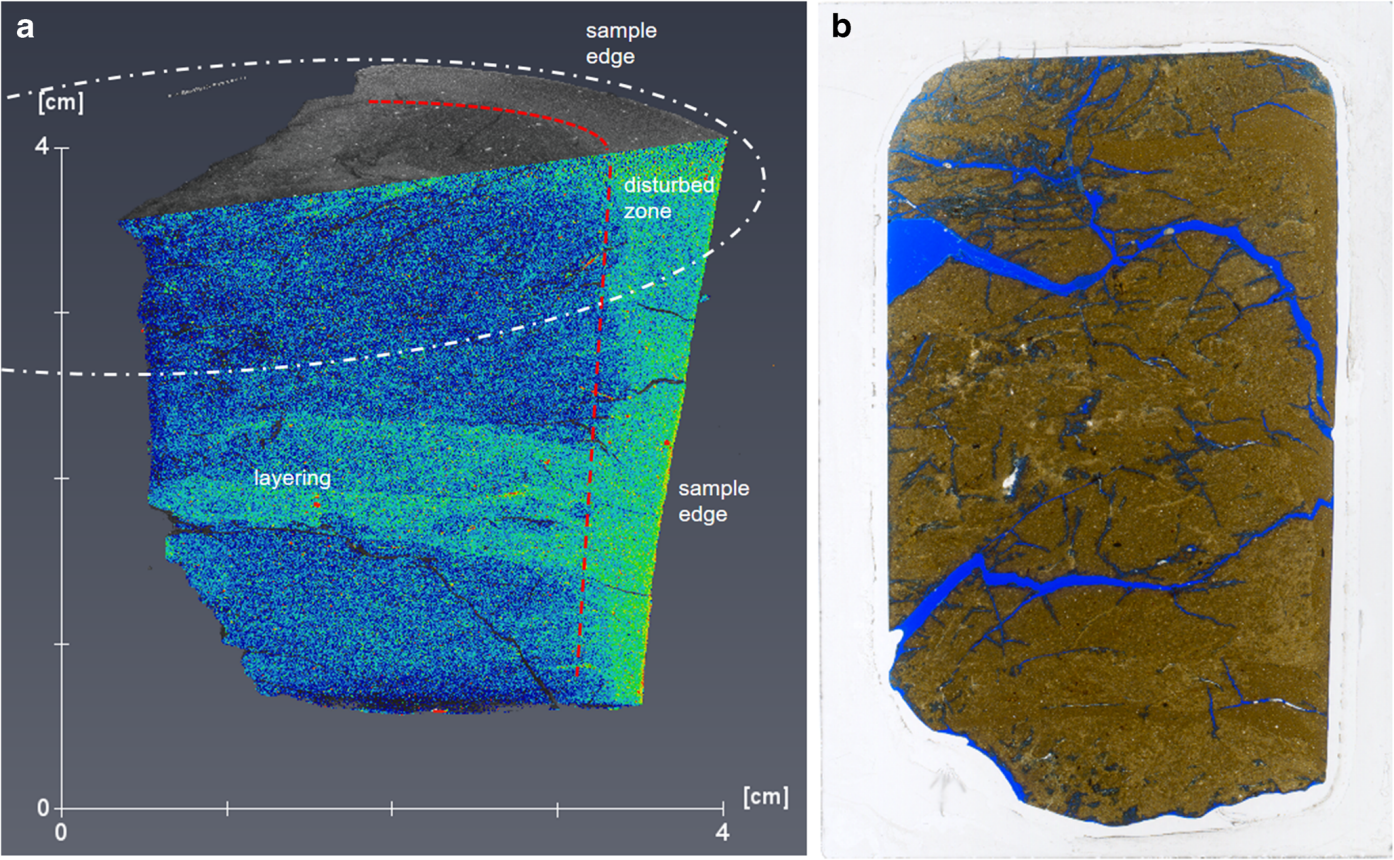
**a** 3D presentation of KM1-B4a. The sample shape represents quarter of a drill core sample (white dashed line represents the original drill core cross section. Top part of the sample is presented in grey scale where lighter colours indicate denser materials. On the vertical cross section, colouring has been used to highlight density differences (from highest to lowest density: red, yellow, green, blue) Most dense accessory minerals are shown in white. **b** Original thin section 78 × 50 mm (way-up ↑) (from Alexander et al. 2017)

Figure 3a shows a snapshot of the 3D image of KM1-B4a together with a thin section example from the same sample location (from Alexander et al. 2017). The larger scale scan of KM1-B4a (Fig. 3a) show very good preservation of the in situ sedimentary layering. In Fig. 3a, disturbance caused by the drilling is very clearly visible in the outer edge of the sample as denser area that has distorted the original mostly horizontal layers. This bending was observed also by Alexander et al. (2017), but the density effect cannot be seen in the thin section. Also, some secondary fracturing is visible (formed due to drying and transport of the samples), but compared with the thin section (Fig. 3b), the disturbance is much less.

**Fig. 4:**
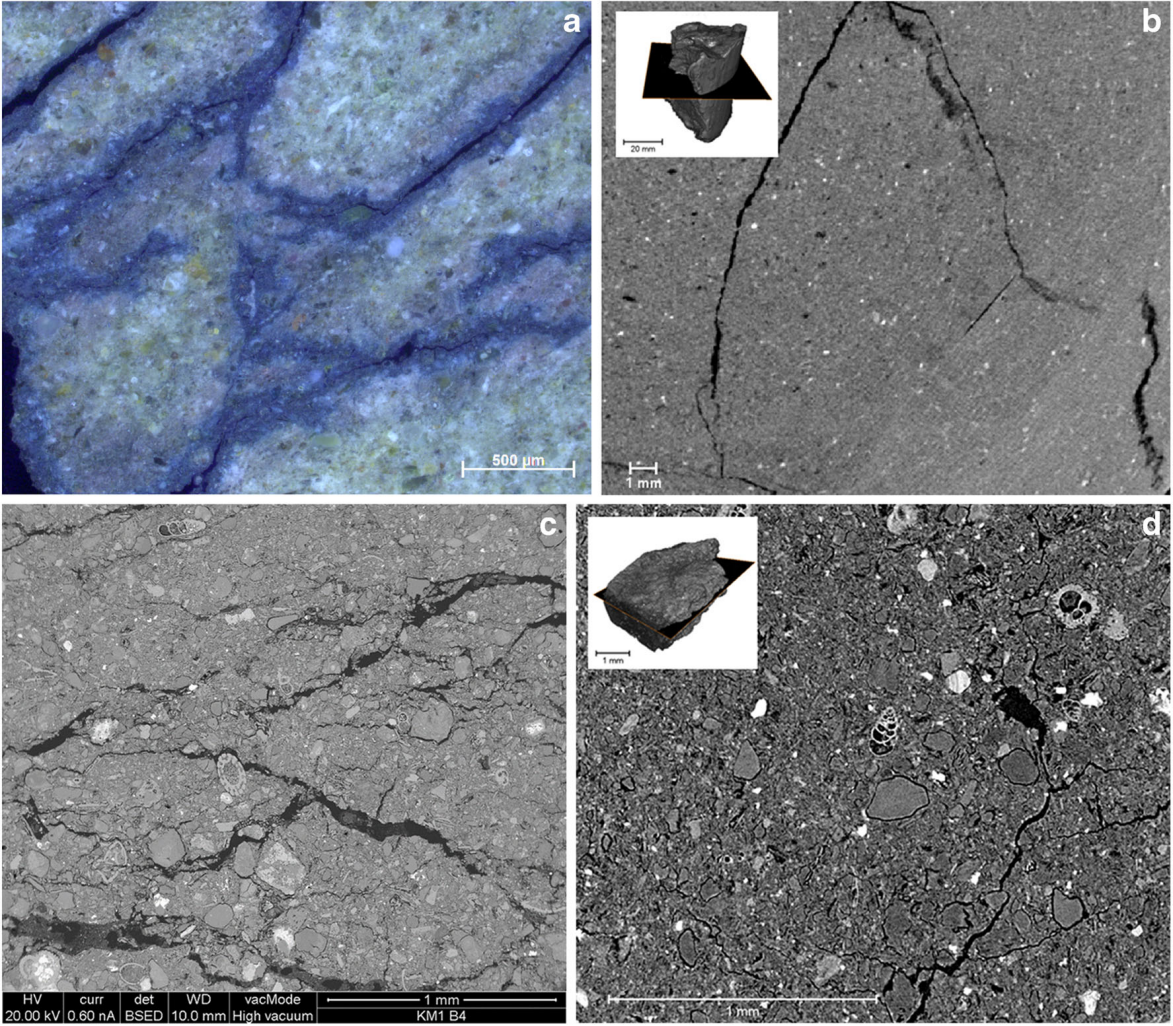
Comparison of the petrographic investigations by Alexander et al. (2017) and XCT scans on samples for KM1-B4. Examples of the cases where non-destructive analysis gives comparable or better information compared with conventional methods. Top, disturbed thin section sample (**a**) and non-destructive XCT (**b**); middle, microfossils show well in thin section (**c**) but can be observed also using XCT (**d**); bottom, granular textures and clast types show well in both thin section (**e**) and XCT (**f**)

Hence, what XCT provides in addition to the original study is less disturbance of the samples. This is especially clear when looking at the porosity increase close to secondary fractures in the blue dye thin sections (Fig. 4a), a feature which is not evident in the XCT image (Fig. 4b). In the XCT image, secondary fractures are clean and cut the bentonite which shows no density increase along the fractures. The apparent higher porosity observed in the thin section (Fig. 4a) is likely caused by the free volume in the thin section sample (total volume increases when secondary fractures are created) and subsequent impregnation with blue dye that has penetrated the bentonite along the secondary fracture edges suggesting that the porosity increase is not a feature of the in situ bentonite (Fig. 4b).

Looking further at the small scale sample KM1-B4b and the original BSEM data (Alexander et al., 2017), again, very similar observations can be made down to features at ~ 100 μm scale by both methods. This is especially evident when observing the microfossils, accessory minerals (denser than matrix) and granular bentonite textures in the KM1-B4b sample (Fig. 4c and d). Propagation pattern of secondary fractures around the granules can be clearly observed in both BSEM and XCT analysis. Accepting the fact that XCT has a lower resolution than BSEM, for example, and needs to be supported by mineralogical analysis, it is clear that, as a quick scanning tool, it provides a way to obtain insight into the material structure and fine features before sub-sampling for further analysis. More importantly, it records the in situ texture, including potential evidence of sampling artefacts (deformation, density changes), which can be later used to assess disturbances caused by the further sample handling and thin-section making procedures.

**Fig. 5:**
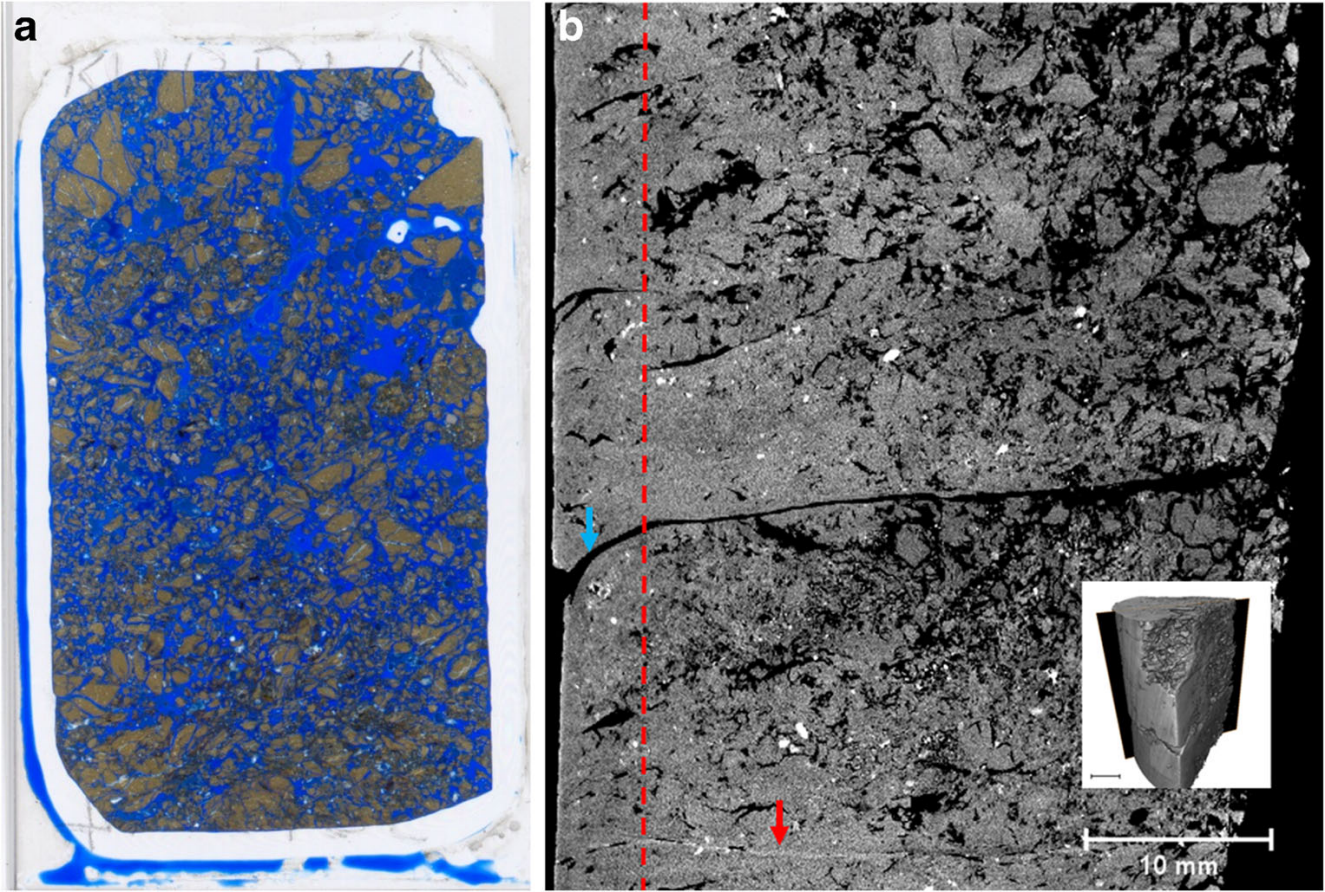
Sample KM3-B1 examples illustrating **a** the disturbance caused by thin section making in the case of porous sample material and **b** cross-section of 3D model (shown in bottom right) showing preservation of in situ textures in the sample

As an example of less intact natural bentonite material, sample KM3-B1 was taken from a bentonite quarry exposure close to the surface. Similar angular texture is observed in both thin section by Alexander et al. (2017) and the XCT scan (Fig. 5), but the original texture is thoroughly perturbed in the thin section (Fig. 5a), leading to loss of any information of sedimentary layer structures that can be observed in the XCT image (Fig. 5b). As a note to the previous interpretation of the geological evolution (cf. Alexander et al. 2017), it seems that the sample might be a result of a disturbed bentonite by quarrying or bentonite erosion and slip down the slope, considering its angular structure.

Overall, the XCT observations from Kato Moni drillhole samples show that preservation of in situ textures and structures in less dense bentonite samples can be significantly improved by including the XCT analysis before further examination.

### Phase II: Tsukinuno and Dobuyama samples

Here, the goal was to assess the internal structure of different types of bentonite sampled in the Tsukinuno mine, classified by their hand specimen appearance as sandy, massive and waxy. To the naked eye, all these bentonites have distinct fabrics. At Tsukinuno, the bentonite grade varies (smectite content 35–80% reported by the mining company, KIC) the higher grades being comparable with that of prefabricated bentonite blocks (e.g. 75–90% reported by Posiva 2012 for buffer bentonite surrounding the waste). The Dobuyama sample differs from the Tsukinuno samples in having a distinctive lapilli tuff texture.

**Fig. 6:**
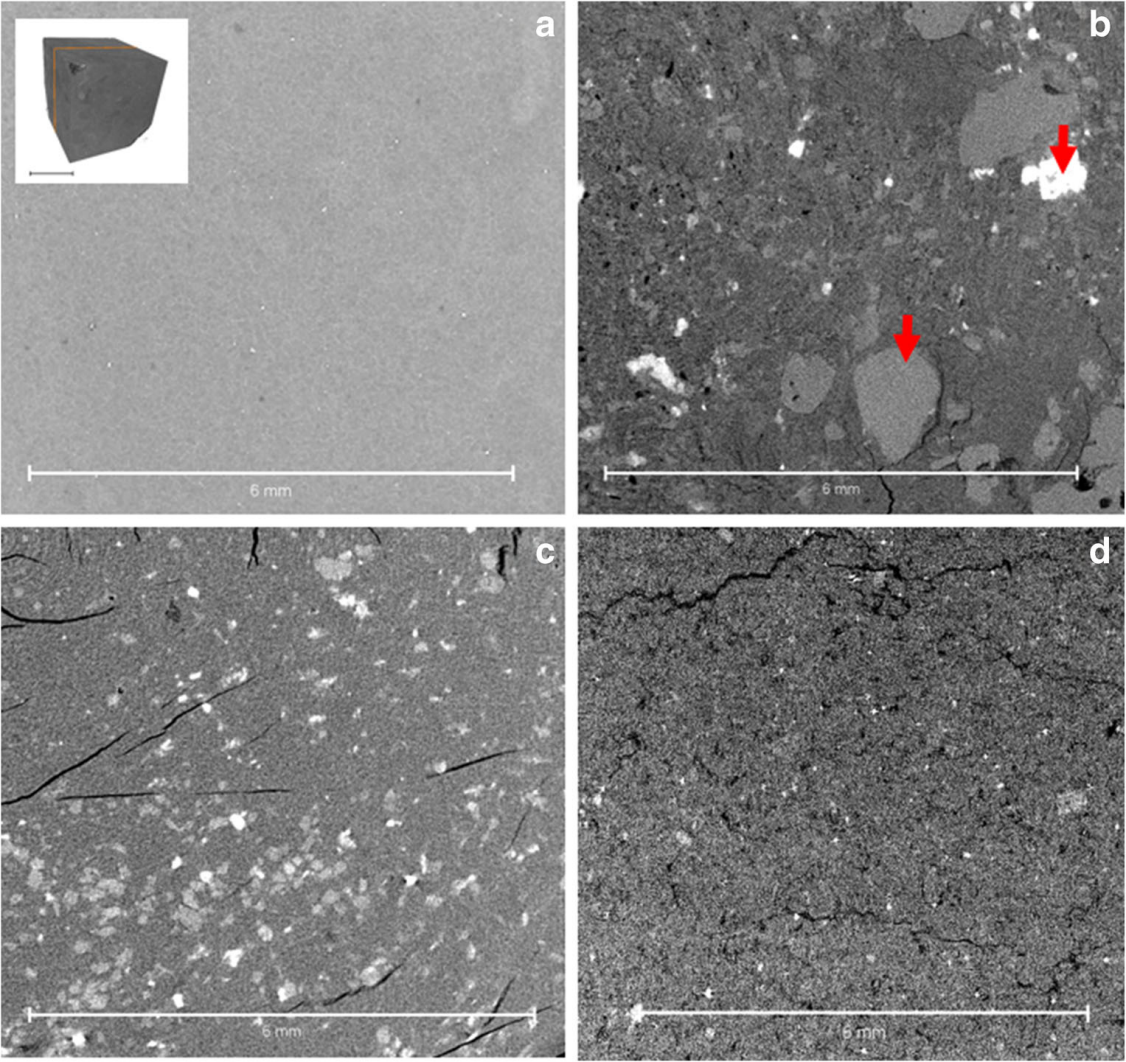
Cross sections of 3D XCT images (example shown in top left corner) of **a** massive, **b** sandy and **c** waxy bentonites from Tsukinuno mine and **d** precompacted bentonite block. Denser materials (accessory minerals) show in lighter colour, while void spaces and fractures can be seen as black. Differences in overall darkness arise from illumination settings. In all samples, the mass is composed of clay, accessory clasts show as lighter, angular or subangular clasts

**Fig. 7:**
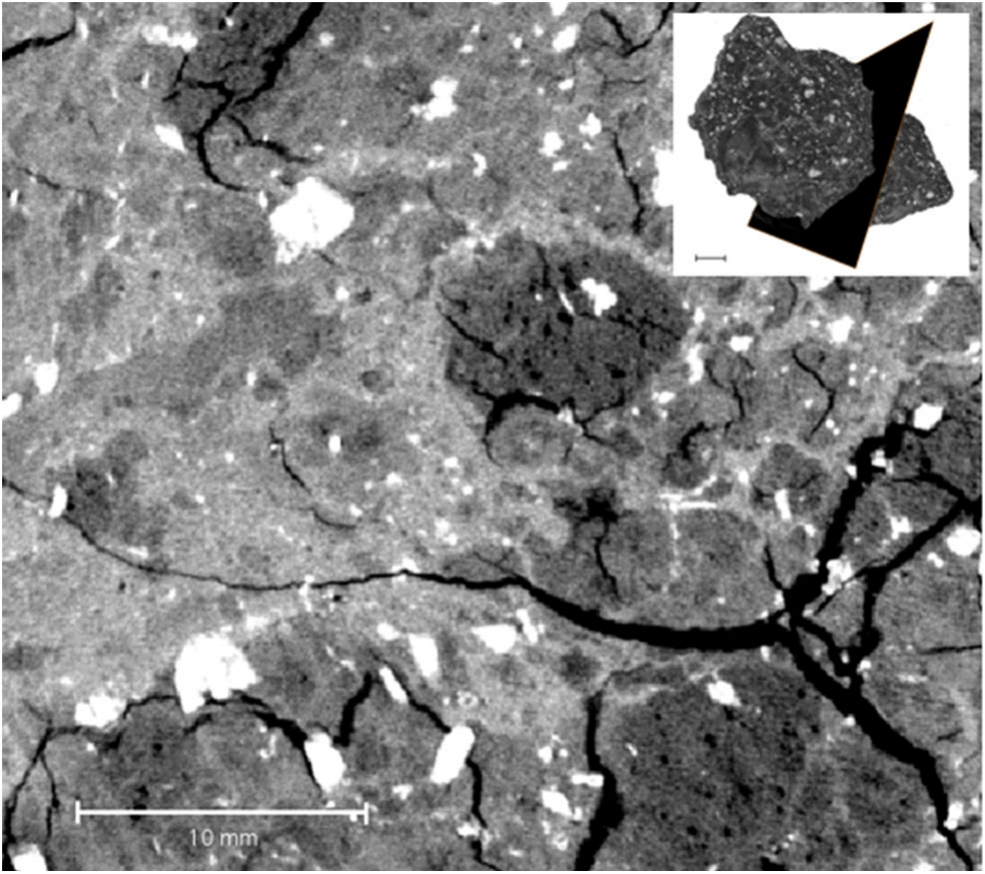
Cross section of 3D XCT image (shown top right) of the pelletal bentonite from Dobuyama. Both scale bars are 10 mm wide. Darker and lighter grey clay areas are clearly visible. White angular clasts are accessory minerals. Fracturing is secondary due to drying of the sample. The darker areas have more micro fracturing, showcasing the initial density difference visible also as small voids

The massive natural bentonite sample shows a very uniform and homogenous texture throughout the sample, both regarding any denser accessory minerals or void spaces (Fig. 6a). Sandy (Fig. 6b) and waxy (Fig. 6c) natural bentonites show more internal heterogeneity at the selected scale. A compacted bentonite sample is shown for comparison in Fig. 6d (see Section 5.4 for discussion). The pelletal texture in the Dobuyama sample is also clearly observed using XCT (Fig. 7). Tsukinuno samples have relatively uniform composition, while the Dobuyama bentonite has two compositionally different clay components (light and dark, in hand specimen, opposite in XCT images), these differences are readily observed also with XCT (Fig. 7: see section 5.4).

### Phase III: compacted bentonite and pellet wetting experiment

**Fig. 8:**
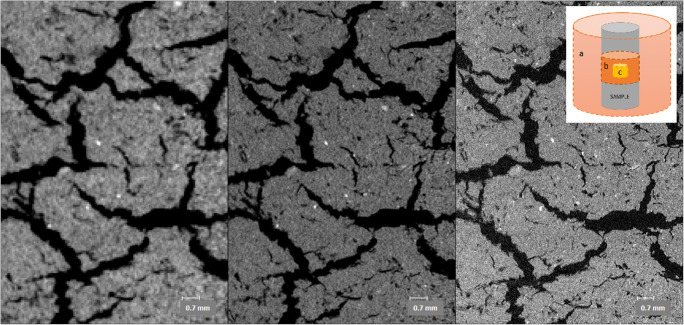
Block 1 imaged at different resolutions and fields of view, comparing the same area from each scan: left (block 1a): whole sample (Ø ~ 5 cm, length ~ 12 cm), voxel size 64.15 μm, scan time 13 min, middle (block 1b): region of interest scan (5 cm of sample imaged), voxel size 30.15 μm, scan time 45 min, right (block 1c): region of interest scan 2 (1.6 cm of sample imaged), voxel size 10.38 μm, scan time 180 min

The block sample was selected to examine block boundary structures in the experimental set up where the boundary area of two prefabricated bentonite blocks were sampled by drilling a cylinder shaped sample (which was held inside a plastic vacuum bag before and during scanning) (see Fig. 3). The XCT scan from the block sample very clearly shows the massive intact bentonite (Fig. 6d) but also internal structure of the void spaces between the original bentonite blocks (Fig. 8). This sample was taken from an unsaturated block structure, and resulting from this, a drying pattern, typical for clays, is observed along the block boundary. The drying pattern shows a continuous void structure. This structure is visible at all resolutions, but the total observed porosity increases when using a higher resolution (Fig. 8). This is important to note in case the aim is to calculate the total porosity.

**Fig. 9:**
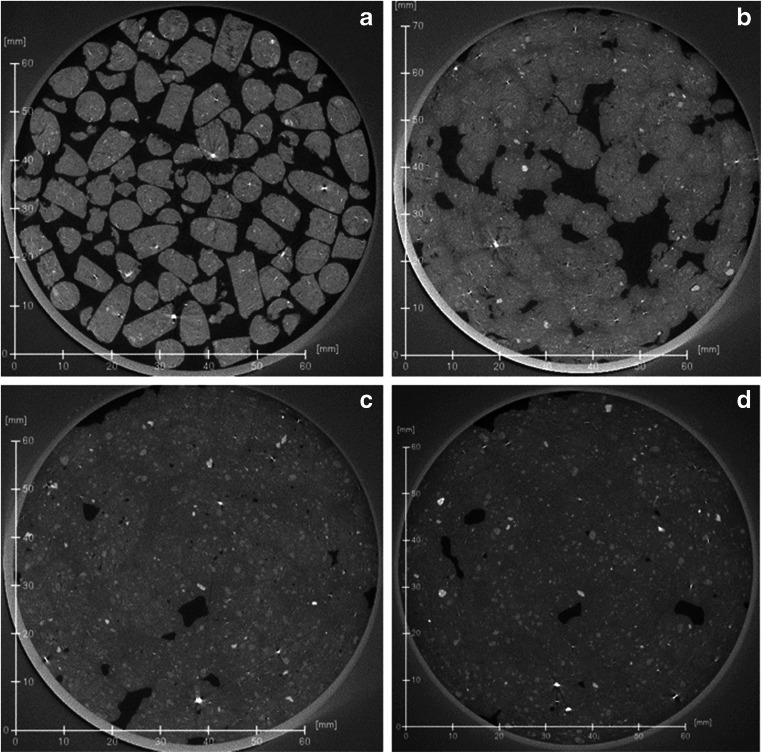
Temporal evolution of relative density differences in wetted bentonite pellets. **a** Dry pellets. **b** After 47 h (partial wetting). **c** After 7 days (partial saturation after 2nd wetting). **d** After 60 days (after 3rd wetting). Wetting was done in steps, which led to uneven swelling and gaps in the bentonite mass. At 60 days, some of the added water was still on top of the bentonite mass as a separate layer

In addition to static samples, XCT was also applied in a simple dynamic experimental set-up in a pellet wetting experiment to test the applicability of the method (Fig. 9). It is acknowledged, that one experiment does not provide sufficient information for process understanding (which would need larger experimental matrix and sample characterization), but as a proof of concept, it was deemed useful. Here, different scan times were applied initially (Fig. 9a), at initial saturation phase (Fig. 9b), and periodically after the experiment reached more stable conditions (Figs. 9c and d) (cf. Molinero Guerra 2018, where water was applied continuously). The XCT 3D images allow observation of movement of the accessory minerals during the swelling process and provide insight to the homogenisation process. Only one type of bentonite pellets was used in this experiment and it would be interesting to explore the behaviour of different pellet types. The main observation from the experiment was that discontinuous application of water slows down the saturation and can lead to air pocket formation during initial phases (observations made up to 60 days in this experiment). Full saturation of the bentonite at the bentonite water interface also retards the water uptake in the bentonite as a whole in the given experimental conditions. In the resulting tomography model, density differences show clearly, especially in early phases of the saturation. Accessory minerals in pellets seem to remain in place while clay particles fill the initial void spaces in between due to the swelling in contact with water. Porosity and related hydraulic conductivity changes have been observed by Sarkar and Sumi (2016) in XCT study on compacted bentonite-sand samples. As noted above, further investigations are needed to provide a better insight into the wetting process, but it is evident, as also shown by Molinero Guerra (2018) that XCT imaging can be applied to wetting experiments for initial state description, changes during experiment and post-mortem analyses.

### XRD and XRF results

Additional analysis for phase II samples were obtained by XRD, to provide background information on the samples (see existing data and reference in Table 1 for Kato Moni samples). Qualitative XRD (Table 6) results from the investigated samples show that Tsukinuno bentonites contain smectite and quartz, and some clinoptilolite, calcite and pyrite as accessory minerals (similar to previously reported by Takagi 2005). Dobuyama sample is mineralogically different, as expected, showing strong peaks of smectite and some accessory plagioclase (lighter bentonite) and plagioclase, quartz and zeolite (potential clinoptilolite) as accessory mineral. The swelling properties of the smectite phase was measured by XRD from oriented clay preparations, and all samples were confirmed to contain smectite-group clay as the only clay phase. For the Dobuyama bentonite, smectites in the light and dark sub-samples indicate differences in smectite structure. These samples were further investigated via semi quantitative XRF. Based on the bulk chemical composition, a systematic compositional difference is observed (two analyses from both samples). Lighter bentonite contains more Al, Fe, Mg and Ca than the dark sample. The dark sample appears more felsic in composition, resembling rhyolitic composition containing more Si, Ti, Na and K when compared with the light sample. The light sample is almost pure smectite, while the dark one also contains some quartz, plagioclase and zeolite. Mineralogy reported here is consistent with the interpretation of Takagi (2005) as a rhyolitic lapilli tuff parent rock, but it is important to notice that the colour differences in nearly pure natural bentonite might still retain differing chemical compositions that probably reflect the primary volcanic texture.

**Table 6:** XRD results for Tsukinuno and Dobuyama samples. Smectite (S), quartz (Q), plagioclase (P), calcite (C), pyrite (PY), zeolite (ZEO), clinoptilolite (CL)

Sample	Interpreted mineralogy	Reference for diffractograms
T1	S + Q + cl + c + py	Figures 1, 2 and 3 in Online resource 2
TM1	S + Q + p + c + py	Figures 4, 5 and 6 in Online resource 2
TW1	S + Q + c + p + py	Figures 7, 8 and 9 in Online resource 2
D2_light	S + pl	Figure 10 in Online resource 2
D2_dark	S + pl + qz + zeo	Figure 10 in Online resource 2

## Discussion

### Usability of XCT in textural/structural characterization

As the phase I results show (Kato Moni samples), XCT scans can provide invaluable information, in addition to existing data (Alexander et al. 2017), on bentonite textures that would be easily deformed by immersion procedures (such as in thin section preparation) due to the plastic properties and swelling of the material. In the original study (Alexander et al. 2017) on the Kato Moni bentonites, thin sections were carefully prepared, however, based on the XCT examination presented here, even the epoxy resin applied in vacuum used to minimize swelling (see details in Section 3.3 in Alexander et al. 2017), still causes extensive disturbance to the samples (see Figs. 3 and 4). In addition, using the blue dye to assess porosity changes might be slightly misleading, since the largest porosities tend to focus around secondary fractures, in the areas, where there is void space to bentonite to expand (see Fig. 4a and b). The disturbance caused by the preparing of the thin section can be distinguished from original textural and structural features, but using XCT prior to thin sections could remove uncertainties significantly as there would be bench-mark for the area selected for the thin section. In addition, phase I results clearly show deformation of the drill core edges due to drilling as an increased density and rotational patterns (curving of the structures close to drill core edge). Less secondary fracturing was observed in the XCT samples, even after storing them for 2 years more than the thin sections made for Alexander et al. (2017). It is also of note that the difference in density between the accessory minerals and the clays is clearly visible in all phase I samples. In principle, this means that the XCT image can be used to calculate their volume in the sample if mineralogical analyses to specify the accessory mineral types are available and the density differences between phases are large enough. In case accessory minerals have similar densities, only the total volume can be calculated.

Phase II (Tsukinuno and Dobuyama) and phase III (EBS) samples that are of higher bentonite quality, in terms of smectite content and general homogeneity, than Kato Moni samples were also proven to benefit from XCT imaging; textural details were observed (Figs. 6, 7, 8 and 9). In general, since a 3D model is produced, observing structural features becomes much easier compared with any 2D analysis. Directions of bedding or fault planes could be easily measured from the XCT model, but for deposit scale investigation, samples should be taken preserving the orientation. The wetting experiment confirms that the method is also applicable for dynamic experiments. However, for a full assessment of the saturation and homogenisation process, a larger experimental matrix would be required. There are uncertainties related to the interpretation of the wetting experiment due to limited data. The XCT results should only be used to guide further analytical planning with supporting mineralogical analysis.

Due to XCTs flexibility, it is possible to make subsequent measurements on laboratory experiments and to observe differences at various water contents and pressures. Here, a simple pellet wetting experiment was undertaken, but more complex experiments are equally possible. In addition to the wetting process examination on pellets, in this study, all the natural samples had been allowed to relax, enhancing structural discontinuities and pre-existing structures. A sample from a large scale experiment was investigated in vacuum packaging, providing information on a less disturbed sample. Based on the experience here, using XCT in situ structures can be easily separated of secondary damage, caused by sample handling, drilling or drying, to samples. XCT provides a tool that complements other analytical methods, and it has limitations regarding the minerals with very similar density. However, as a non-destructive method, it is seen as a useful tool to provide an overview on potential textures and structures that can be used to guide further work.

Existing mineralogical data (Tables 1 and 6) help to interpret the XCT images, e.g. separate clays from quartz or feldspar (high density difference). In addition to smectite, natural bentonite samples discussed here commonly contain quartz, plagioclase, calcite (Kato Moni) and quartz (Tsukinuno). In order to produce mode detailed mapping of minerals in XCT images, further mineralogy analyses would be required.

### Comparison of natural and prefabricated bentonites

Three natural bentonites were investigated, Kato Moni (heterogeneous bentonite), Tsukinuno (homogenous bentonite) and Dobuyama (pelletal bentonite). In general, to assess the performance of bentonite based EBS, natural bentonites can be used as analogues (NA) to these prefabricated materials, since they can be found in compositions and conditions that are similar to those expected to prevail in geological repositories (see e.g. Reijonen and Alexander 2015). Depending on the application, in EBS systems variable, bentonite types are planned to be used, including high quality bentonite buffers (e.g. Posiva 2012; SKB 2011), lower quality bentonite backfills (e.g. Posiva 2012) and bentonite-sand or bentonite-aggregate mixtures (e.g. Man and Martino 2009).

Samples in this study cover most of the bentonite types planned to be used in a repository. Based on the samples described in this study, it can be seen that by using XCT, information on the internal heterogeneity or homogeneity of bentonite deposits could be exponentially increased due to significantly faster analysis times compared with conventional destructive methods. This would allow subsequent analyses to be targeted in a more efficient way as well as to allow more direct comparison with specific prefabricated bentonite types. By investigating the internal structures of bentonites, at relevant densities (similar to those of prefabricated bentonites), the relevance of the processes investigated can be better targeted to the engineered system of interest. The more detailed information can also be used to assess the relative importance of a specific *feature* to the *overall performance* of the bentonite material, i.e. the internal variation within a bentonite deposit, may not affect its overall performance at all (e.g. low hydraulic conductivity or radionuclide retention properties can be obtained by bentonites with a variable range of smectite content), information that would bring additional robustness to the repository designs or enlarging the safety envelope.^2^ As such, XCT does not provide information on the hydraulic conductivity itself, but it helps to increase the spatial understanding from point wise measurements to EBS/deposit scale. On the other hand, the method may help in pointing out features that would be missed by point wise measurements (e.g. fracturing), which might have important implications to some other process (e.g. gas transport through bentonite).

In general, based on the limited number of samples examined here, similar textures are observed in natural and prefabricated bentonites at similar densities:

- massive, homogenous bentonites with varying amounts of accessory minerals (samples T1, TW, TM, Block, Pellet, KM1-B4a, KM1-B4a, KM3-B1, KM3-B5)
- bentonite-aggregate mixtures (D1, KM2-B4, KM3-B1)

This provides a good basis to study processes relevant to geological repositories via further NA studies. The samples analysed here were taken randomly from the bulk bentonite and especially for samples from the interfaces (e.g. bentonite-shale at Tsukinuno), XCT can provide valuable insight when studying interface processes, such as potential bentonite expansion to shale fractures, or if textural controls exist for chemical compositions, since any major textural or density change could be readily observed. Regarding the physical similarity of the studied clays, it seems that, in this particular case, the massive natural bentonite seems even more homogenous (less accessory minerals) than the prefabricated MX-80 (block), supporting understanding that very similar materials to prefabricated bentonites can be found in nature.

What is of additional interest in the MX-80 block sample in the broader sense is that if in case bentonite blocks are not fully saturated, the gaps could be preserved along the original block boundaries, meaning that gas transport (in particular) through the bentonite would likely to occur along the block boundaries in the continuous void space, rather than through the bentonite bulk materials, that is seen in saturated bentonite, as demonstrated in experiments such as large scale migration test in Grimsel underground rock laboratory in Switzerland (e.g. Shimura et al. 2006). This process would be of interest in cases where bentonite based geomaterial has experienced drying.

## Conclusion

The XCT data obtained in this study is able to provide valuable information on the textural patterns observed in all the samples analysed.

Overall, compared with thin section analysis, XCT measurement is fast and can be performed in minutes to few hours (Table 2), even when using high resolution scans. It provides 3D data which holds great benefits in assessing overall sedimentary and microstructures of the bentonite samples. The XCT method and its usability for petrographic characterization of bentonite materials have great potential in bentonite investigations, both for bentonite deposits and experimental research. Depending on the equipment, XCT provides detection of important physical features of bentonite at various scales, including internal textures, structures, density variation, porosity and void spaces. Sedimentary structures in bentonites are well visible due to density changes; even using 2D X-ray imaging would help in the selection of sampling locations for natural bentonite samples providing easier/faster continuous description of the samples at deposit scale. 3D imaging can provide information on the structural planes and their orientation and estimates of the accessory mineral volumes (needs verification points by quantitative analyses). In addition to original features of the samples, drilling (or sampling) artefacts are clearly visible (denser areas and apparent bending at the edge of the core, fracturing by drying etc.). By sampling carefully, these artefacts can be avoided; samples can be analysed in a container (e.g. plastic), enabling textures to be preserved as in situ. Sampling related disturbances are of increased importance especially in applications where density is an important parameter. XCT also provides an effective tool to bench mark sample conditions prior sub-sampling e.g. for thin section making. All this is of importance when studying geomaterials which functioning in environmental solutions is based on physical integrity and properties that provide the desired retention and sealing properties for chemical substances.

From the samples analysed here, no full evaluation of differences between natural and prefabricated bentonites can be made, but based on the examples presented XCT, assessing the relevance of the analogy between natural and processed bentonites can be greatly improved by implementing XCT and more detailed petrographical analyses on bentonite investigations (e.g. density, textures and composition variation within natural vs. prefabricated bentonites). In addition to application of XCT in research, XCT could also be further developed as a non-destructive method for quality control applications of industrial processes, such as bentonite components production for geological repositories (e.g. scanning of bentonite blocks for foreign objects or void spaces). Since XCT does not provide mineralogical or chemical information, to have the most benefit of XCT analysis, it has to be complemented it with other mineralogical and petrographic examinations to verify initial observations. It is also acknowledged that, even with assistance from other methods, XCT is not able to distinguish between mineral of similar density.

## Electronic supplementary material

Online resource 1 (PDF 3634 kb)

Online resource 2 (PDF 1173 kb)

## Footnotes

1 “The natural bentonite is usually processed (i.e. beneficiated) to improve the smectite content of the material. It is also usually chemically treated to enhance its cation exchange, swelling or other physico-chemical properties (e.g. conversion or “activation” of natural calcium montmorillonite to sodium montmorillonite by treatment with sodium carbonate). As such, the physical and chemical properties of processed bentonite may differ somewhat from that of the natural rock and this limitation must be borne in mind when considering the results.” (Milodowski et al. 2016)

2 According to use by IAEA, the safety envelope represents that set of safety boundary conditions within which the geological disposal facility has to perform, throughout its life cycle, in order to comply with the legal and regulatory requirements. The safety envelope should be identified by the operator in the safety case by taking into consideration applicable regulatory requirements. The safety envelope represents an “outer boundary” that must not be violated.
