# Supplementary material for: Benefits of applying X-ray computed tomography in bentonite based material research focussed on geological disposal of radioactive waste
Source: Environ Sci Pollut Res Int. 2020 Mar 2;27(31):38407–21. doi: 10.1007/s11356-020-08151-2 (PMC7524814; doi:10.1007/s11356-020-08151-2)
Supplement: Supplementary file 1 — (PDF 3634 kb) [file 11356_2020_8151_MOESM1_ESM.pdf]

Online resource 1 – Petrographic comparisons

**Petrographic results from the original study on epoxy impregnated thin sections: Alexander et al. 2017.**	**Selected images of XCT analysis on untreated bentonite samples (taken adjacent to original samples)**
**Sample KM1-B4**	
Sample KM1-B4 (depth): 9.60-9.70 m (along drillhole) (large format thin section 78x50 mm)	Sample KM1-B4a and b (depth): 9.70-9.80 m (along drillhole)
**Optical description:**<br>*“Two major sub-horizontal fractures cross cut the section, from which numerous, angular, minor fractures originate (Plate C 24). The fractures appear clean with no evidence of secondary precipitation. Blue dye has penetrated some distance into the matrix on either side of the fractures indicating a slight increase in porosity surrounding the fractures (Plate C 25). The matrix is dense with some slight variations in density indicated under reflected light. Some fossil fragments and micro-fossils are present within the matrix (Plate C 26).” (Alexander et al. 2017)*	**Comparison to optical analysis**<br>Overall, the sample is more intact than the thin section sample, despite of some additional fracturing due to transport and storage of the samples for about 3 years. In the XCT scan, the deformation of the sample around the edges due to drilling is evident (higher density in the lighter areas around the edge of the drill core section, see right edge in Figure A1 and close to round edge in Figure A2.
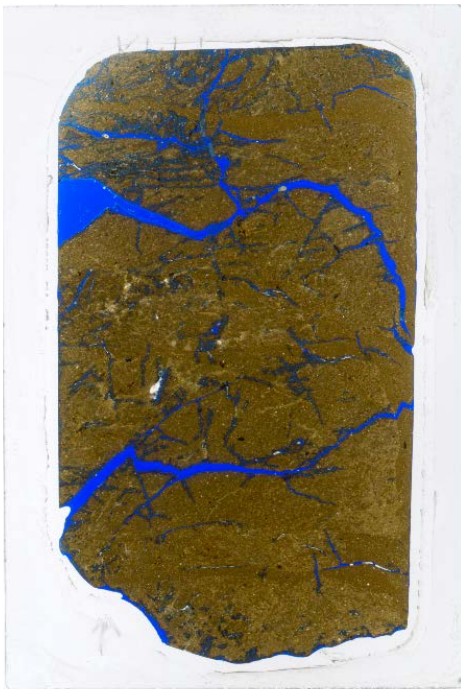<br>Plate C 1 Scanned image of the thin section (way-up ↑). Samples size: 78x50 mm.<br>(Alexander et al. 2017)	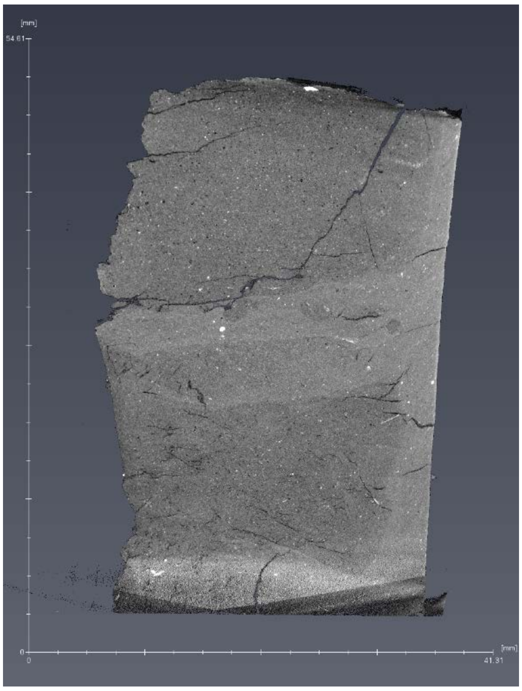<br><br>Figure A1. KM1-B4a. Less fracturing and no disturbance due to the epoxy impregnation. Way up (↓). Density differences show layering structure. Accessory minerals visible.

<table>
<tr><th>Petrographic results from the original study on epoxy impregnated thin sections: Alexander et al. 2017.</th><th>Selected images of XCT analysis on untreated bentonite samples (taken adjacent to original samples)</th></tr>
<tr><td></td><td>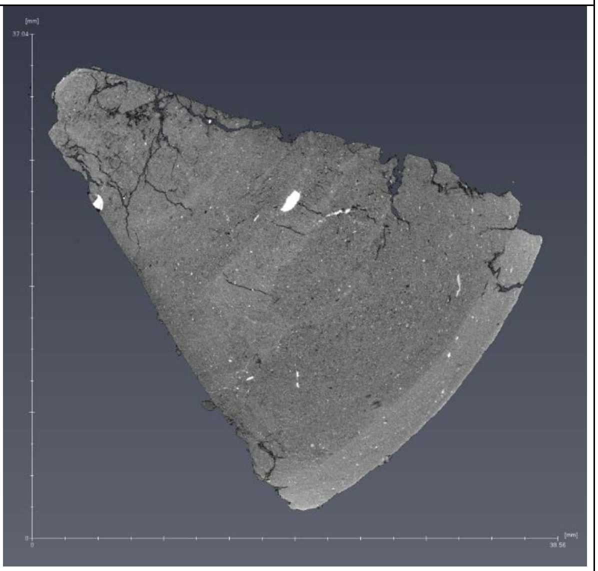
Figure A2. KM1-B4a. Image taken from top down (horizontal section). Deformation caused by drilling is visible as denser area along the rounded drill core edge (visible effect < 5 mm from sample edge). Also other density differences within the sample can be seen. Bright accessory minerals show clearly.</td></tr>
<tr><td>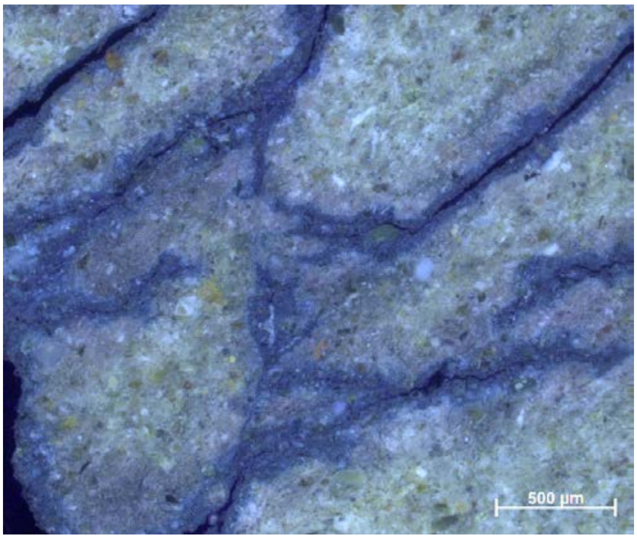

Plate C 2. Optical microscopy. Dark-field image showing part of the fracture network, showing the extent of blue-dye penetration either side of a fracture indicating a slight increase in porosity within the proximity of a fracture. (Alexander et al., 2017)</td><td>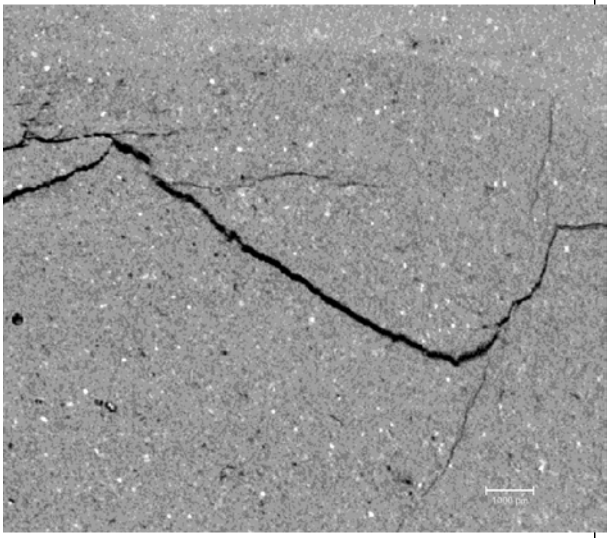
Figure A3. KM1-B4a. Snapshot taken from the XCT 3D model (KM1-B4a sample). Porosity change observed as impregnation of the dye is likely to be artefact due to immersion of the dye in unconfined conditions. Note the scale difference. Samples for XCT were not dyed. In this type of cases, non-destructive XCT analysis prior to any further sampling and thin section preparation would provide invaluable information regarding the origin of porosity features (i.e. are they original properties or artefacts).</td></tr>
</table>

<table>
<tr>
<th>Petrographic results from the original study on epoxy impregnated thin sections: Alexander et al. 2017.</th>
<th>Selected images of XCT analysis on untreated bentonite samples (taken adjacent to original samples)</th>
</tr>
<tr>
<td>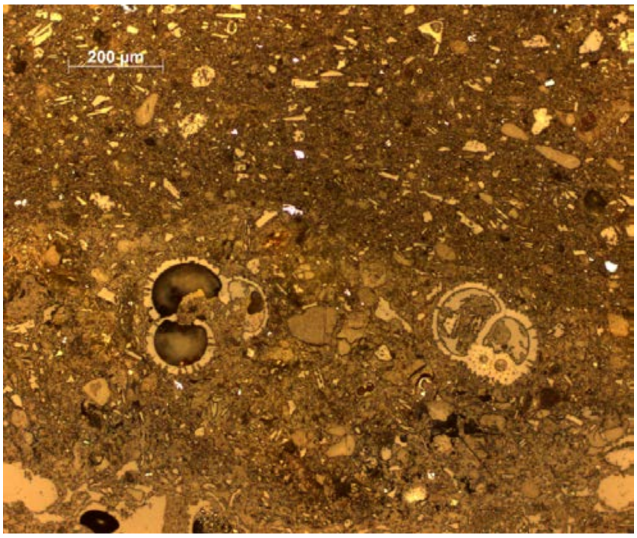
Plate C 2. Reflected light image showing the dense nature of the matrix, some micro-fossils (foraminifera), and a slight change in matrix density between the upper part and the lower part of the image. (Alexander et al. 2017)</td>
<td>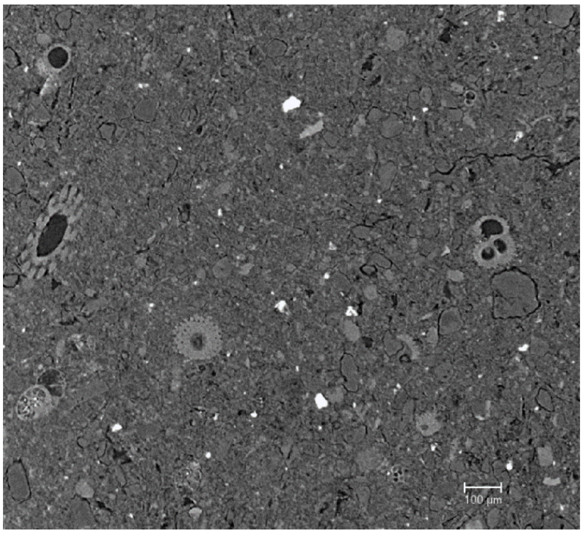
Figure A4. Snapshot taken from the XCT 3D model (KM1-B4b) in a similar scale as Plate C 26 on the left. Microfossils and granular texture of the bentonite are clearly visible, almost comparable to the reflected light image of this section (see left). Differences in the minerals with similar densities are more difficult to see.</td>
</tr>
<tr>
<td>SEM analysis
“The BSEM images reveal the extent of the fracturing which is pervasive throughout the sample (Plate C 4). In general, fracturing has propagated around the coarser grains: very few are themselves fractured. No evidence of secondary precipitation was observed. Some differences in porosity were observed: areas of finer grained material appear less porous than coarser areas. These differences in finer grained and coarser grained areas reflect the original sedimentary deposition textures (Plate C 5). “ (Alexander et al. 2017)</td>
<td>Comparison to SEM analysis
KM2-B2b with high resolution XCT from a small sample provides similar resolution to petrographical observations as BSEM images. XCT images also provide this information in 3D format if needed, providing new way of assessing structural features, such as orientation. Samples studied here were not oriented.</td>
</tr>
</table>

<table>
<tr><th>Petrographic results from the original study on epoxy impregnated thin sections: Alexander et al. 2017.</th><th>Selected images of XCT analysis on untreated bentonite samples (taken adjacent to original samples)</th></tr>
<tr><td>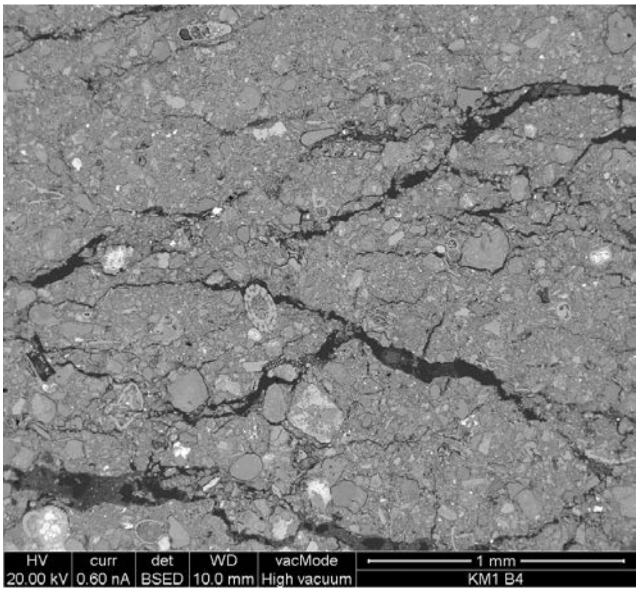

Plate C 3. BSEM image showing the nature of the fracturing: very few of the larger grains are themselves fractured, the fractures have largely propagated around these coarser grains. (Alexander et al. 2017)</td><td>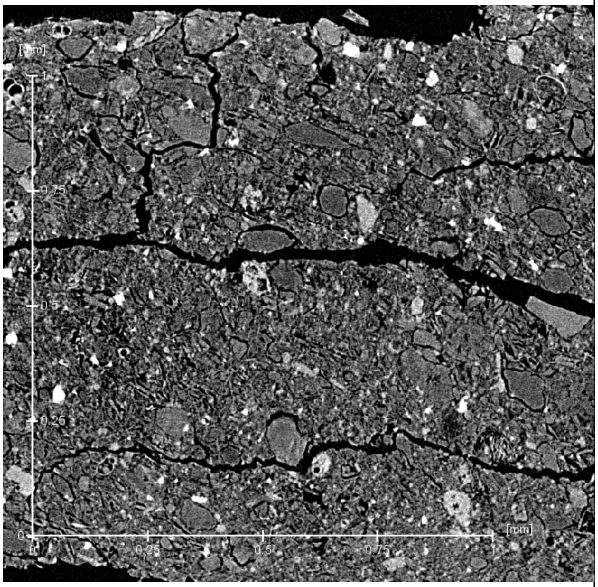

Figure A5. KM1-B4b. Similar secondary fracturing visible in the high resolution XCT sample (note the slight scale difference). Also granular texture and accessory minerals are detectable.</td></tr>
<tr><td>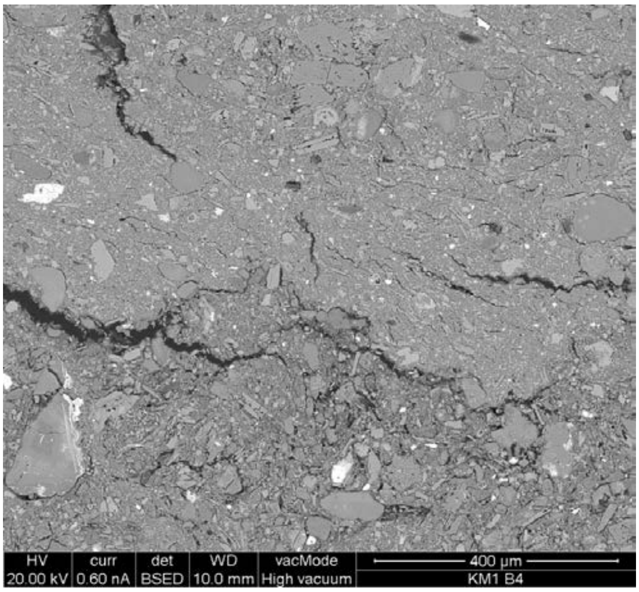

Plate C 4. BSEM image showing some differences in texture: upper portion has a lower porosity than lower half of image, and is finer grained. This reflects differences in the original sediment deposition. (Alexander et al. 2017)</td><td>Figure A1 and A2 show clear density differences in a larger scale. Small scale differences was not observed in the selected high resolution sample KM1-B4b (small sample <5 mm in diameter) (Figures A4 and A5).</td></tr>
<tr><td colspan="2">Sample KM1-B7</td></tr>
<tr><td>Sample KM1-B7 (depth): 11.55-11.61 m (along drillhole) (normal size thin section 48x28 mm)</td><td>Sample KM1-B7 (depth): 11.61-11.66 m (along drillhole)</td></tr>
<tr><td>Optical analysis</td><td>Comparison to optical analysis</td></tr>
</table>

Petrographic results from the original study on epoxy impregnated thin sections: Alexander et al. 2017.	Selected images of XCT analysis on untreated bentonite samples (taken adjacent to original samples)
“The matrix is dense and fossiliferous. There is a network of fractures across the sample dominated by horizontal / sub-horizontal fractures. Some bands within the section are highly porous. Pre-existing bedding features have influenced the fracturing to some extent e.g. propagation along very fine grained seams, or fracturing around more dense ‘clots’ – palletisation.” (Alexander et al. 2017)	

Petrographic results from the original study on epoxy impregnated thin sections: Alexander et al. 2017.	Selected images of XCT analysis on untreated bentonite samples (taken adjacent to original samples)
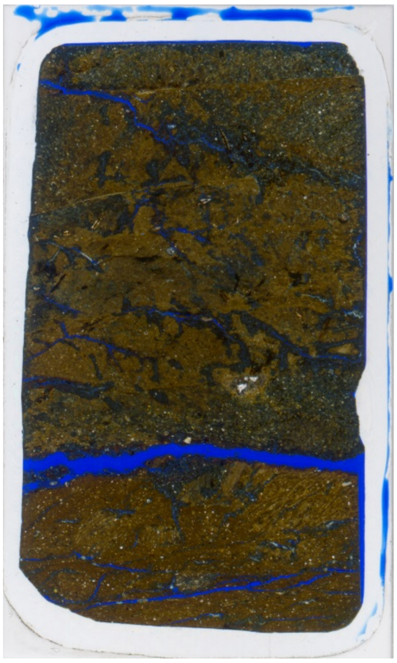<br>Plate C 5. Scanned image of the thin section (way-up ↑). Sample size 48x28 mm.<br>(Alexander et al. 2017)	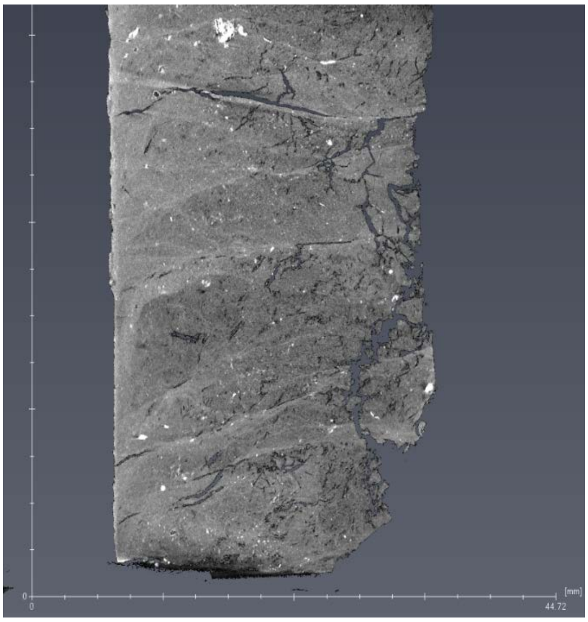<br>Figure A5. Snap shot of the analysed sample (left: the outer edge of the drillcore), way up (↑)<br>- clear indications of deformation on the edge of the sample → drilling artefact<br>- sedimentary layering, secondary fractures follow bedding planes<br>- secondary fracturing (but less than in epoxy sections)<br>- areas with different porosities show clearly (also observed in thin section Plate C 43).
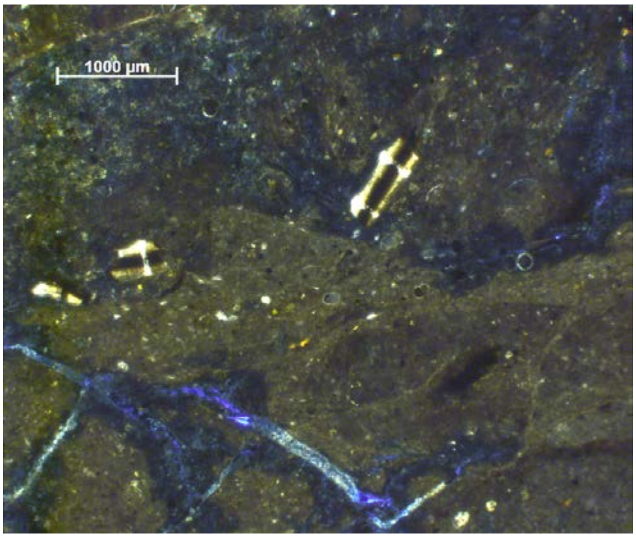<br>Plate C 6. PPL image showing contrast between areas of lower porosity (bottom) and higher porosity (top). Propagation of fractures have been controlled to some extent by bedding features.<br>(Alexander et al. 2017)	See Figure A5, for similar observation at larger scale in XCT.
**SEM analysis**<br>Imaging revealed intense micro fracturing throughout the sample. The dominant orientation of the micro fractures	Micro-fractures are not visible in the resolution of the XCT measurement in this sample. However, the

Petrographic results from the original study on epoxy impregnated thin sections: Alexander et al. 2017.	Selected images of XCT analysis on untreated bentonite samples (taken adjacent to original samples)
changes markedly from sub-horizontal to sub-vertical within differing areas of the section (Plate C 8). There is minor pelletisation of the sample in some areas, where micro fractures have propagated around packets of bentonite with differing grain size or density. No evidence for secondary precipitation other than some minor Fe mobilisation.	resolution used in sample KM1-B4b, would be high enough to observe similar structures.

<table>
<tr>
<th>Petrographic results from the original study on epoxy impregnated thin sections: Alexander et al. 2017.</th>
<th>Selected images of XCT analysis on untreated bentonite samples (taken adjacent to original samples)</th>
</tr>
<tr>
<td>
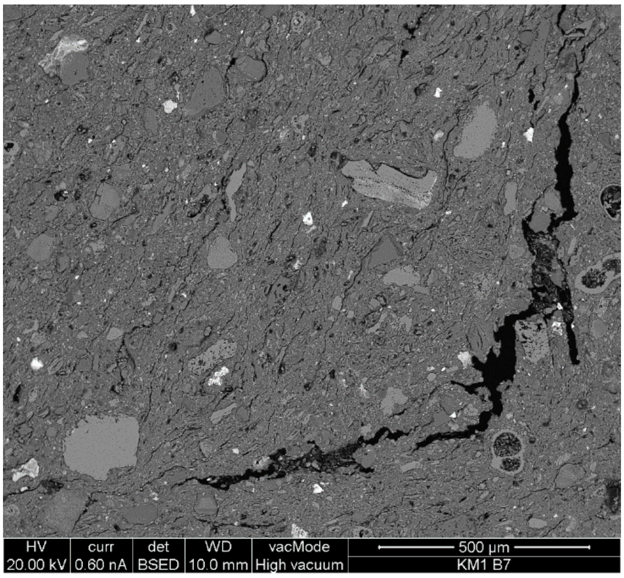

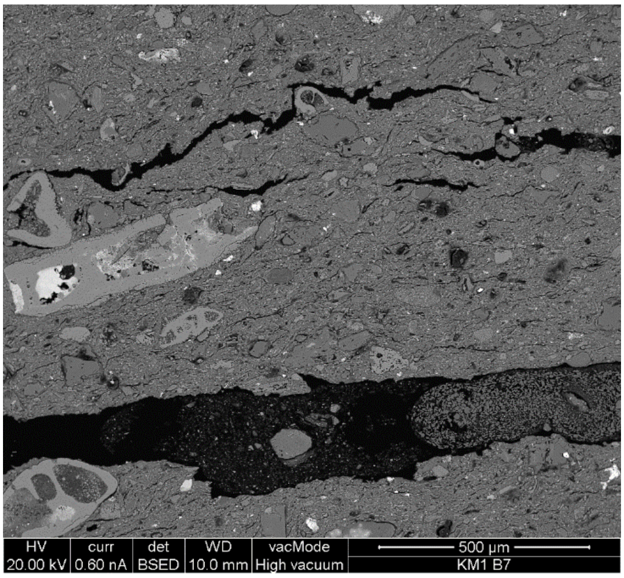

Plate C 7. BSEM images showing the change in fracture orientation between two areas of the sample. Top: micro fracturing is sub-vertical, bottom: micro fracturing is sub-horizontal. (Alexander et al. 2017)
</td>
<td>
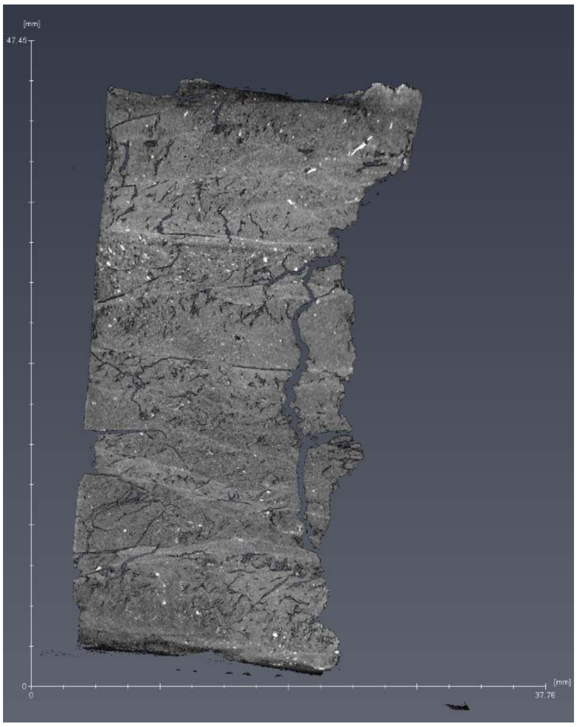

Figure A6. Micro fracturing observed in Plates C44 (left) is not observed at the selected scale of measurement, however, sedimentary layers show clearly. Fracturing (secondary) is clearly observed, including the different orientations and frequency variation between different layers. Horizontal fracturing (through the sample) follows pre-existing layer structures.
</td>
</tr>
</table>

<table>
<tr><td>Petrographic results from the original study on epoxy impregnated thin sections: Alexander et al. 2017.</td><td>Selected images of XCT analysis on untreated bentonite samples (taken adjacent to original samples)</td></tr>
<tr><td>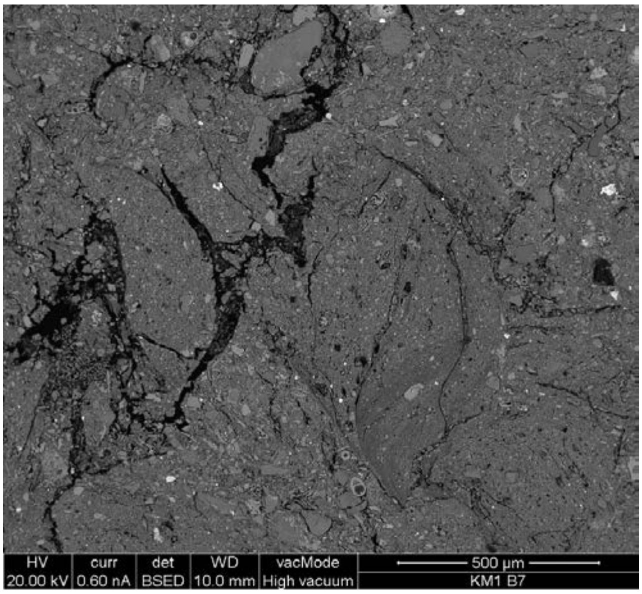

Plate C 8. BSE image showing pelletisation by micro fractures propagating around areas of differing grain size and / or density. (Alexander et al. 2017)</td><td>Pellet type structures are not observed at the selected resolution of the analysis.</td></tr>
<tr><td colspan="2">Sample KM2-B4</td></tr>
<tr><td>Sample KM2-B4 (depth): 14.50-14.65 m (along drillhole) (large format thin section 78x50 mm)</td><td>Sample KM2-B4 (depth): 14.65-14.80 m (along drillhole)</td></tr>
<tr><td>Optical observations
Matrix is rich in micro fossils (Plate C 11). The thin section shows three distinct, evenly distributed, sub-horizontal banding comprising of a finer-grained matrix which is denser and lower in porosity (Plate C 12). Many of the micro-fractures have an en-echelon texture. No evidence for secondary precipitation is present.</td><td>Comparison to optical analysis</td></tr>
</table>

<table>
<tr><th>Petrographic results from the original study on epoxy impregnated thin sections: Alexander et al. 2017.</th><th>Selected images of XCT analysis on untreated bentonite samples (taken adjacent to original samples)</th></tr>
<tr><td>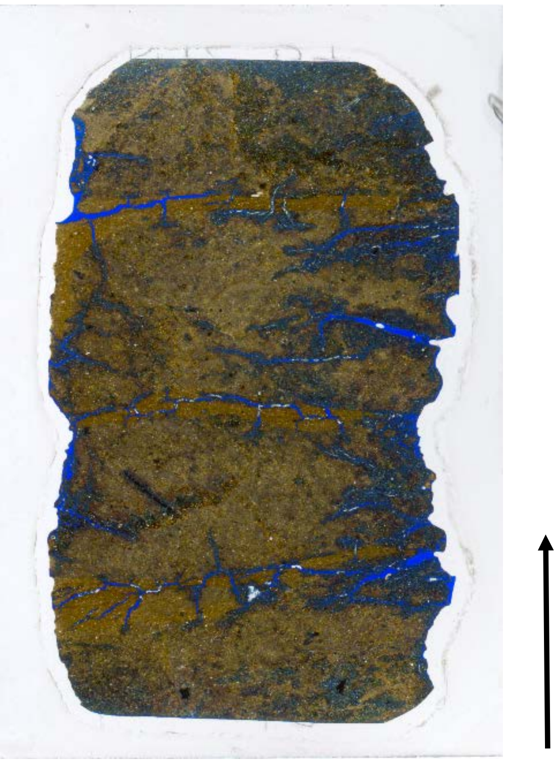
Plate C 9. Scanned image of the thin section showing way-up orientation. Sample size 78x50 mm (Alexander et al. 2017)</td><td>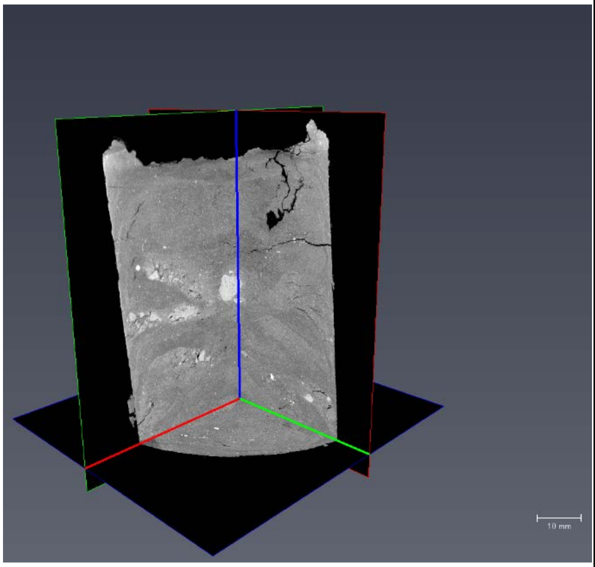
Figure A7. Bedding planes visible also in this sample (as in Plate C 53). In this sample also bigger angular clasts are observed. Some bands are denser than others, similarly to observation in Plate C55.
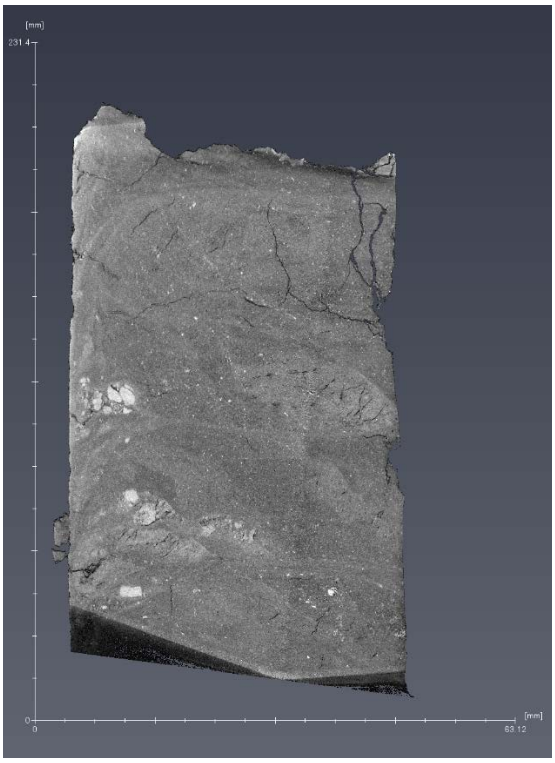

Figure A8. Very similar overall texture is visible as in the thin section. In denser (lighter colour) bentonite rounded clast there are also fractures that could be described as en-enchelon.
This sample also seems to have similar listric type faulting as in sample KM3-B5.</td></tr>
</table>

**Petrographic results from the original study on epoxy impregnated thin sections: Alexander et al. 2017.**	**Selected images of XCT analysis on untreated bentonite samples (taken adjacent to original samples)**
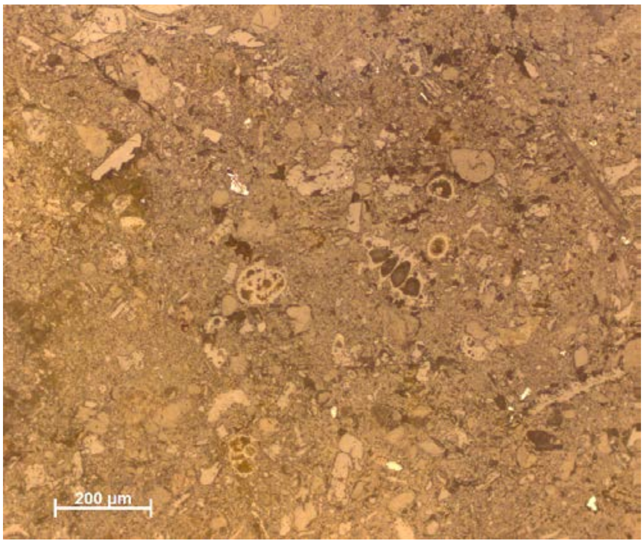<br><br>Plate C 10. Reflected light image showing the fossiliferous nature of the matrix. (Alexander et al. 2017)	In the XCT analysed sample only some microfossils were observed, however, this is due to lower resolution.
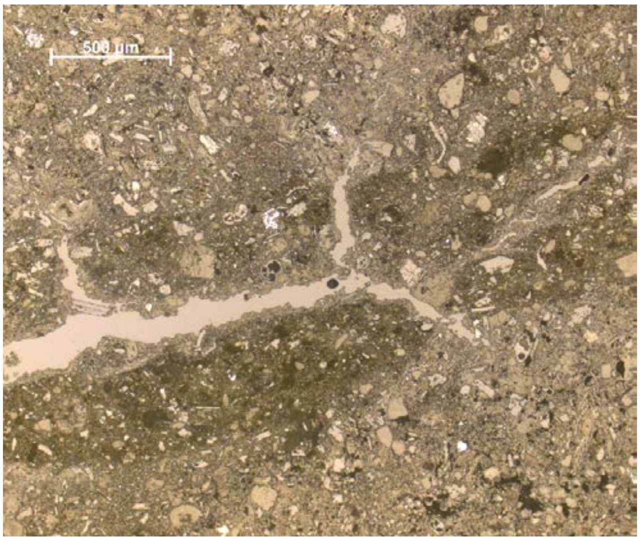<br><br>Plate C 11. Reflected light image showing one of the denser, sub-horizontal bands. The material on either side of the band has a higher porosity. A fracture is present in the centre of the denser band. (Alexander et al. 2017)	
SEM analysis<br>BSEM imaging highlighted the contrast between the dense bands and the more porous matrix. The dense bands are finer grained as shown by Plate C 13. The matrix is fossil rich, containing both fossil fragments and micro-fossils. The matrix is extensively micro fractured, with the micro fractures propagating around the coarser, more competent grains.<br>(Alexander et al. 2017)	Micro-fractures are not visible in the resolution of the XCT measurement in this sample. However, the resolution used in sample KM1-B4b, would be high enough to observe similar structures.

<table>
<tr><td>Petrographic results from the original study on epoxy impregnated thin sections: Alexander et al. 2017.</td><td>Selected images of XCT analysis on untreated bentonite samples (taken adjacent to original samples)</td></tr>
<tr><td>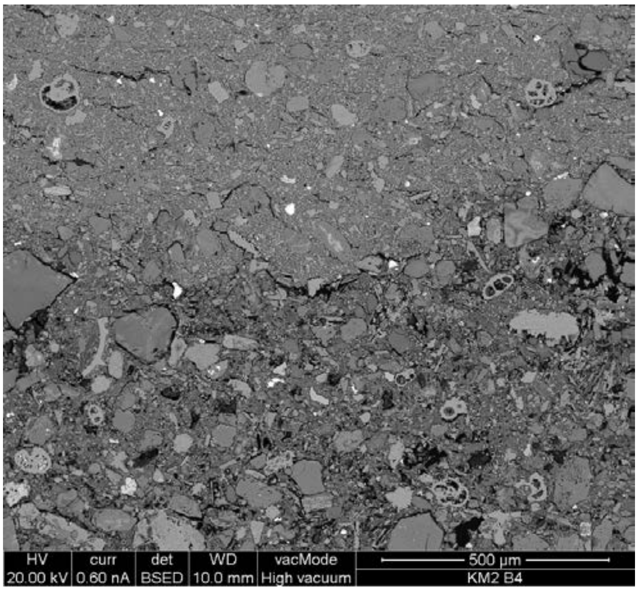

Plate C 12. BSEM image showing the contrast between one of the dense, finer-grained, sub-horizontal bands (top) and the more porous matrix below.
(Alexander et al. 2017)</td><td></td></tr>
<tr><td>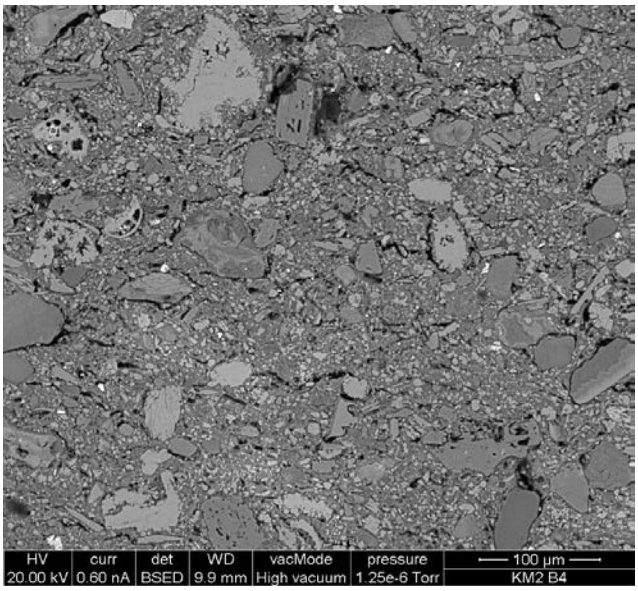

Plate C 13. BSEM image of more porous matrix showing the pervasive micro fracturing. Fracturing has propagated around more competent grains.
(Alexander et al. 2017)</td><td></td></tr>
<tr><td colspan="2">Sample KM3-B1</td></tr>
<tr><td>Sample KM3-B1 (depth): 0.17-0.24 m (along drillhole) (normal size thin section 48x28 mm)</td><td>Sample KM3-B1 (depth): 0.25-0.35 m (along drillhole)</td></tr>
<tr><td>Optical observations
The sample appears pulverised with a network of angular fractures. There is no overriding preferential direction of</td><td>Comparison to optical analysis
XCT preserves sample intact, but shows also the porous nature of the sample. The horizontal fracture in the middle was</td></tr>
</table>

Petrographic results from the original study on epoxy impregnated thin sections: Alexander et al. 2017.	Selected images of XCT analysis on untreated bentonite samples (taken adjacent to original samples)
fracturing. Fossils are rare; the sample is extremely fine-grained. (Alexander et al. 2017)	caused by sample handling. Within XCT sample also sedimentary layering is visible.
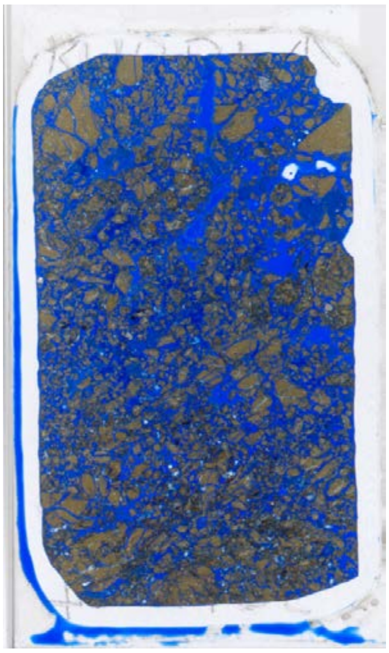<br>Plate C 14. Scanned image of the thin section (way-up ↑). Sample size 48x28 mm. (Alexander et al., 2017). “The sample appears pulverised with a network of angular fractures. There is no overriding preferential direction of fracturing. Fossils are rare; the sample is extremely fine-grained.” (Alexander et al., 2017). Scanned image of the thin section (way-up ↑). Sample size 48x28 mm.	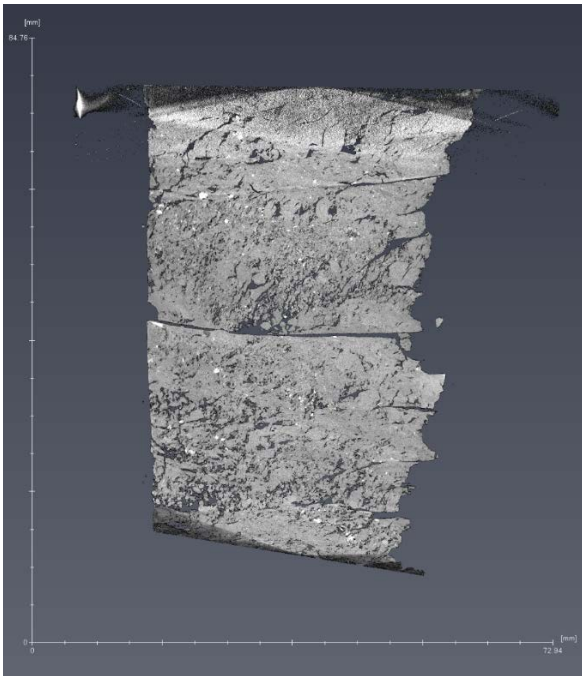<br>Figure A9. The XCT methodology preserves the sample intact, but also shows the porous nature of the sample. The horizontal fracture in the middle was caused by sample handling. Within the sample, the sedimentary layering is also visible. Angular fracturing observed by Alexander et al. 2017 is less evident, but in situ texture has angular clasts. This suggests that angular fracturing is present also in situ.<br>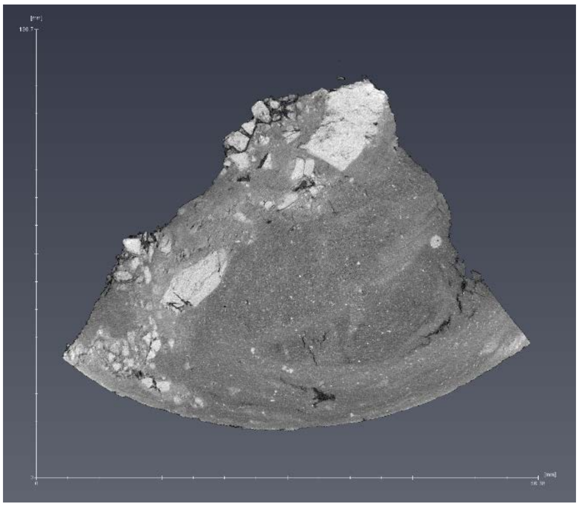<br>Figure A10. Also large clasts of accessory minerals were observed in XCT scan, this can be explained by the slightly different sampling point. The drillcore sample edge seem to be less disturbed than in the samples presented above. This could be due to the denser nature of the sample, as some bending is visible, but no clear density difference can be seen as e.g. in Figure A2.

**Petrographic results from the original study on epoxy impregnated thin sections: Alexander et al. 2017.**	**Selected images of XCT analysis on untreated bentonite samples (taken adjacent to original samples)**
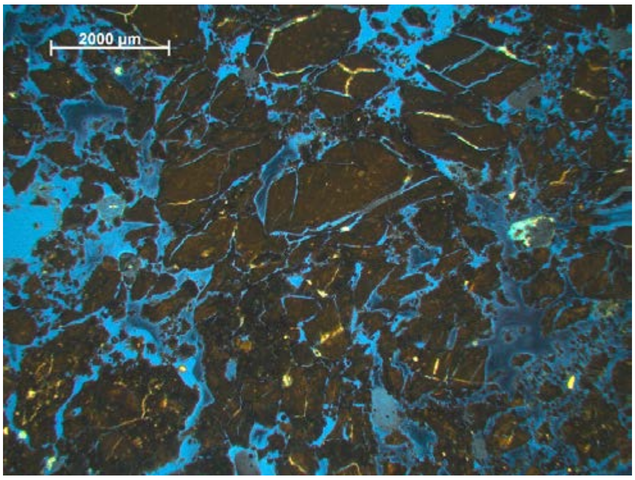<br><br>Plate C 15. PPL image showing the pulverised nature of the sample. The fractures are dominantly angular.	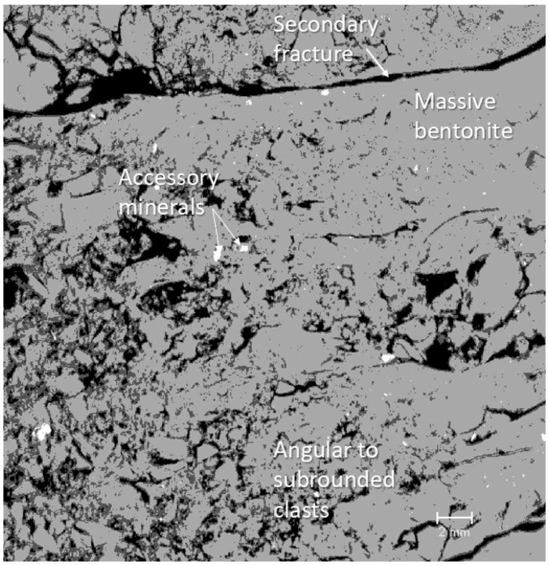<br><br>Figure A11. Close up of sample KM3-B1 XCT model illustrating the angular to sub angular clasts.
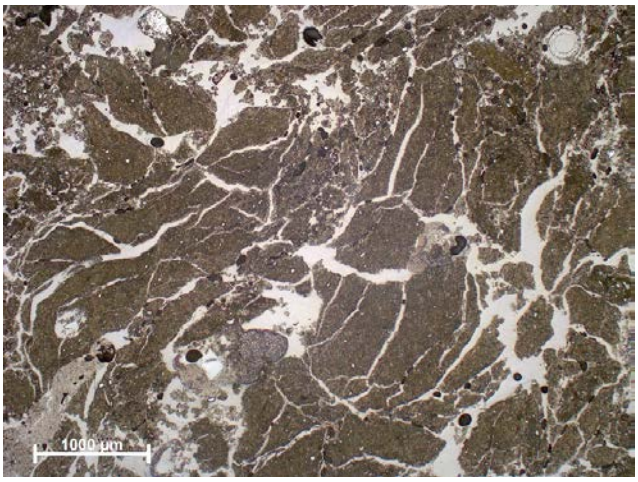<br><br>Plate C 16. Reflected light image showing the extensive nature of the fracturing and the homogenous, fine-grained nature of the bentonite. (Alexander et al. 2017)	See Figure A11.
SEM analysis<br>BSEM imaging highlights the homogenous and fine-grained nature of the bentonite (Plate C 18). Fracturing is extensive and the sample has been pulverised into reasonably equant pieces (Plate C 19). Micro fracturing is present within these pieces (Plate C 18).	Microstructures are not visible in the resolution of the XCT measurement in this sample. However, the resolution used in sample KM1-B4b, would be high enough to observe similar structures.

<table>
<tr><th>Petrographic results from the original study on epoxy impregnated thin sections: Alexander et al. 2017.</th><th>Selected images of XCT analysis on untreated bentonite samples (taken adjacent to original samples)</th></tr>
<tr><td>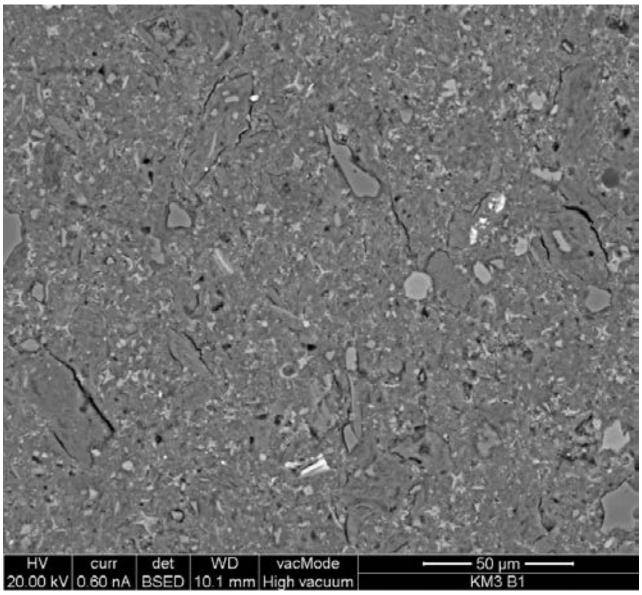

Plate C 17. BSEM image showing detail of the matrix. This is fine grained and contains only rare micro fossils. There is some minor micro fracturing. (Alexander et al. 2017)</td><td></td></tr>
<tr><td>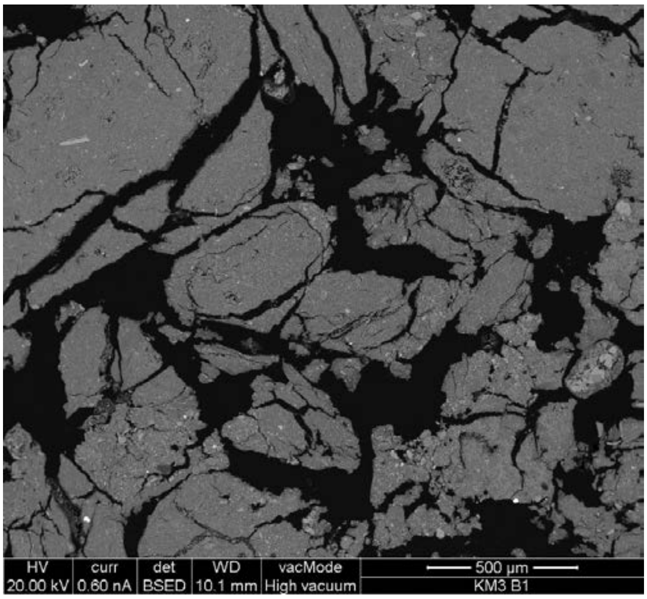

Plate C 18. BSEM image showing the extensive fracturing. (Alexander et al. 2017)</td><td></td></tr>
<tr><td colspan="2">Sample KM3-B5</td></tr>
<tr><td>Sample KM3-B5 (depth): 2.00-2.10 m (along drillhole) (normal size thin section 48x28 mm)</td><td>Sample KM3-B5 (depth): 2.11-2.19 m (along drillhole)</td></tr>
<tr><td>Optical analysis
Numerous listric faults and sigmoidal fractures are visible in the thin section scan (Plate C 20). The resulting clasts are angular to sub-rounded. There are changes in fracture</td><td>Comparison to optical analysis
XCT preserves sample intact, but shows also the porous nature of the sample. In addition, densification of the sample edges can be seen at this resolution.</td></tr>
</table>

**Petrographic results from the original study on epoxy impregnated thin sections: Alexander et al. 2017.**	**Selected images of XCT analysis on untreated bentonite samples (taken adjacent to original samples)**
pattern throughout the thin section, reflecting the localised shear strain (Plate C 21). Some limited development of a pelleted or ‘chicken-wire’ texture is present, with some pellets being clearly defined by curved fractures which contrast to the dominant angular fractures throughout the sample (Plate C 22). Towards the top left of the section, a ‘polka-dot’ texture is present with numerous small light coloured spheres (<100 µm) visible (Plate C 23). (Alexander et al. 2017)	
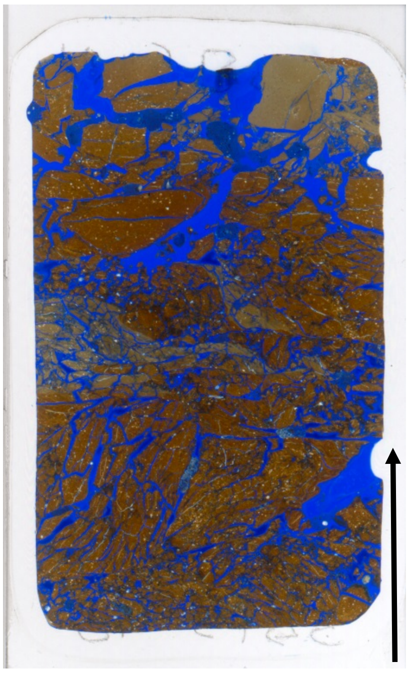 Plate C 19. Scanned image of the thin section showing way-up orientation. Sample size 48x28 mm. (Alexander et al. 2017)	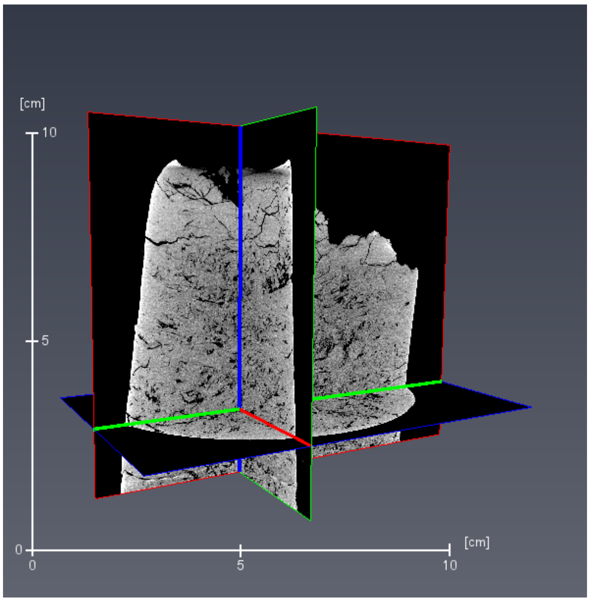 Figure A12. Image of a 3D model of the sample KM3-B5. 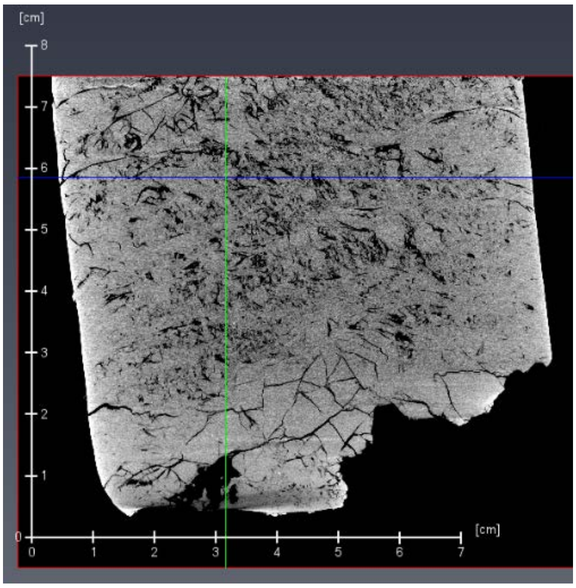 Figure A13. Cross section of the sample KM3-B5 showing the internal porosity of the sample.

**Petrographic results from the original study on epoxy impregnated thin sections: Alexander et al. 2017.**	**Selected images of XCT analysis on untreated bentonite samples (taken adjacent to original samples)**
	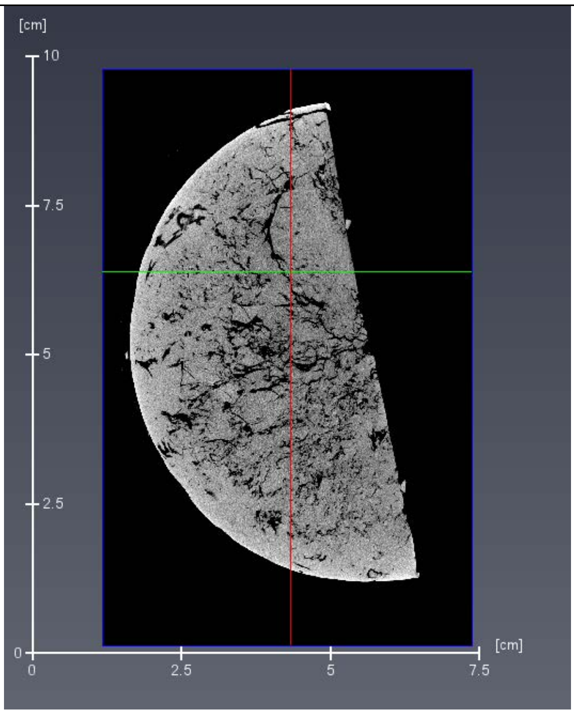<br>Figure A14. Low resolution scan of sample KM3-B5 show higher porosity of this sample clearly. The edges of the sample are slightly denser (white rims on the drill core outer edge). It is likely that drilling has disturbed the outer edge of the sample.<br>Also angular fracturing is visible. XCT preserves the original texture better than epoxy impregnated samples.<br>Curved fractures are observed at cm scale, e.g. in Figure A14, starting at the cross of the red and green lines towards top-left.

**Petrographic results from the original study on epoxy impregnated thin sections: Alexander et al. 2017.**	**Selected images of XCT analysis on untreated bentonite samples (taken adjacent to original samples)**
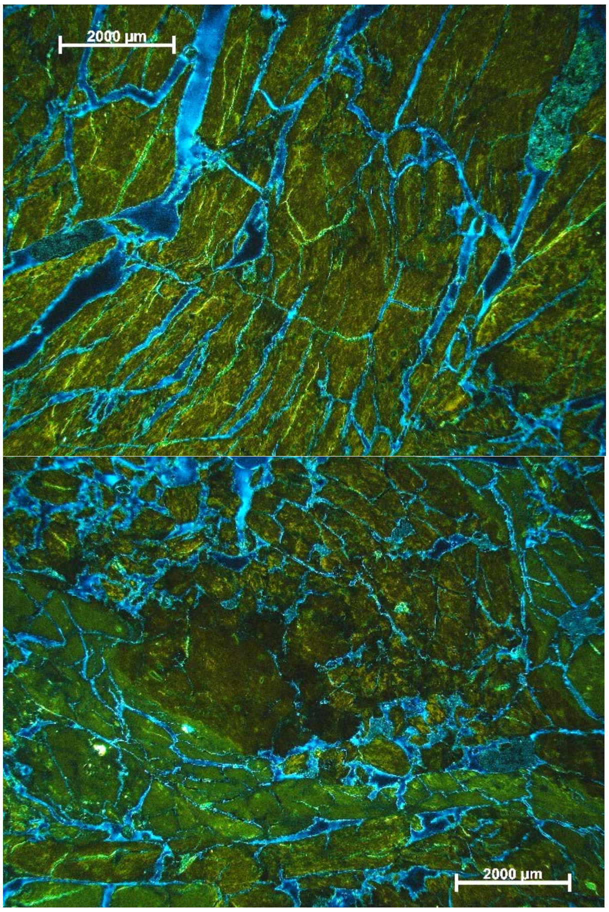<br>Plate C 20. PPL images showing two examples of different localised fracture patterns. Left: the fracturing is dominated by regular, elongate, sub-vertical fractures, whereas right: the fractures are shorter and more sub-horizontal dominated. (Alexander et al. 2017)	See Figure AX. Similar granular structure is seen in XCT.
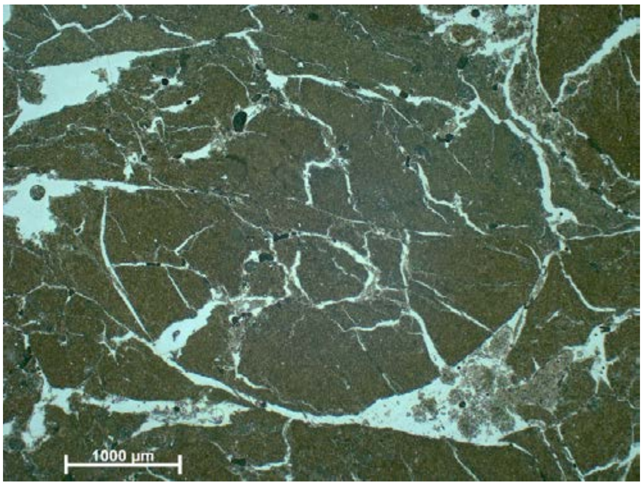<br>Plate C 21. Reflected light image showing a curved fracture defining a pellet. 'Pelletisation', or development of a 'chicken-wire' texture is present but minor in this sample.	

**Petrographic results from the original study on epoxy impregnated thin sections: Alexander et al. 2017.**	**Selected images of XCT analysis on untreated bentonite samples (taken adjacent to original samples)**
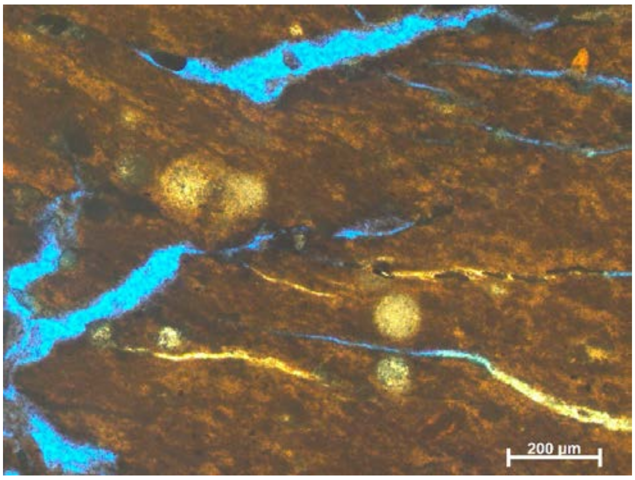<br>Plate C 22. PPL image of the ‘polka-dot’ texture present in the top-left of the thin section. (Alexander et al. 2017)	
SEM analysis<br>The bentonite is extremely fine grained (grains <20 µm) and micro fossil-free. The polka-dots are clusters of Si-rich rims with porous centres (Plate C 24, Plate C 25). These features are probably a diagenetic texture resulting from the breakdown of bacteria or micro-organisms, and are probably a zeolitic composition. Micro fractures have propagated around these polka-dots, indicating that the rims are more competent than the surrounding bentonite. (Alexander et al. 2017)	Microstructures are not visible in the resolution of the XCT measurement in this sample. However, the resolution used in sample KM1-B4b, would be high enough to observe similar structures.
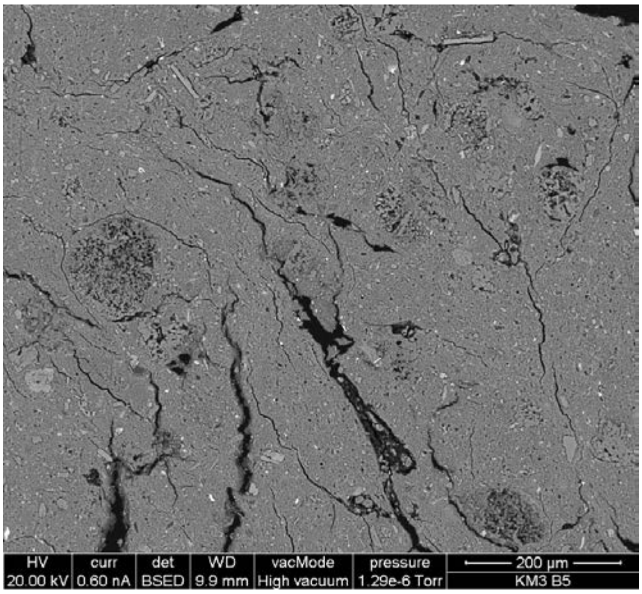<br>Plate C 23. BSEM image of the ‘polka-dot’ texture showing the ‘polka-dots’ with their Si-rich rims and porous centres. Note the propagating of the micro fractures around the ‘polka-dots’. (Alexander et al. 2017)	

<table>
<tr><th>Petrographic results from the original study on epoxy impregnated thin sections: Alexander et al. 2017.</th><th>Selected images of XCT analysis on untreated bentonite samples (taken adjacent to original samples)</th></tr>
<tr><td>
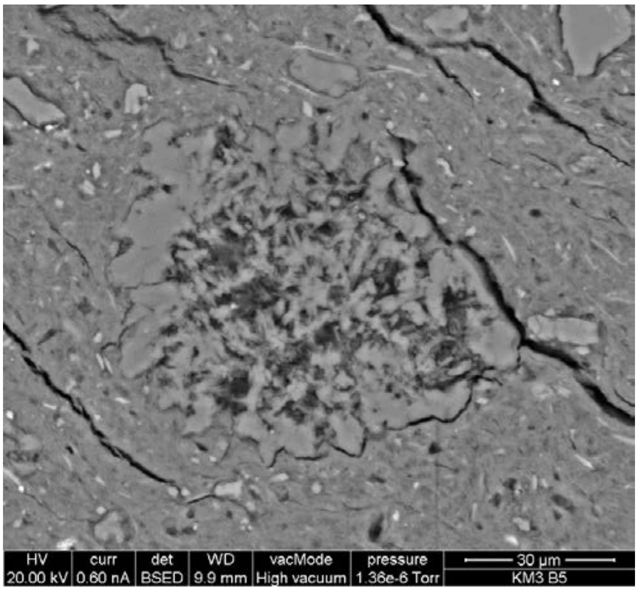

Plate C 24. BSEM image showing a ‘polka-dot’ in detail. The Si-rich rim and porous centre are clearly visible. Note the pathway of the propagating micro fractures. (Alexander et al. 2017)
</td><td></td></tr>
</table>
