# Supplementary material for: Benefits of applying X-ray computed tomography in bentonite based material research focussed on geological disposal of radioactive waste
Source: Environ Sci Pollut Res Int. 2020 Mar 2;27(31):38407–21. doi: 10.1007/s11356-020-08151-2 (PMC7524814; doi:10.1007/s11356-020-08151-2)
Supplement: Supplementary file 2 — (PDF 1173 kb) [file 11356_2020_8151_MOESM2_ESM.pdf]

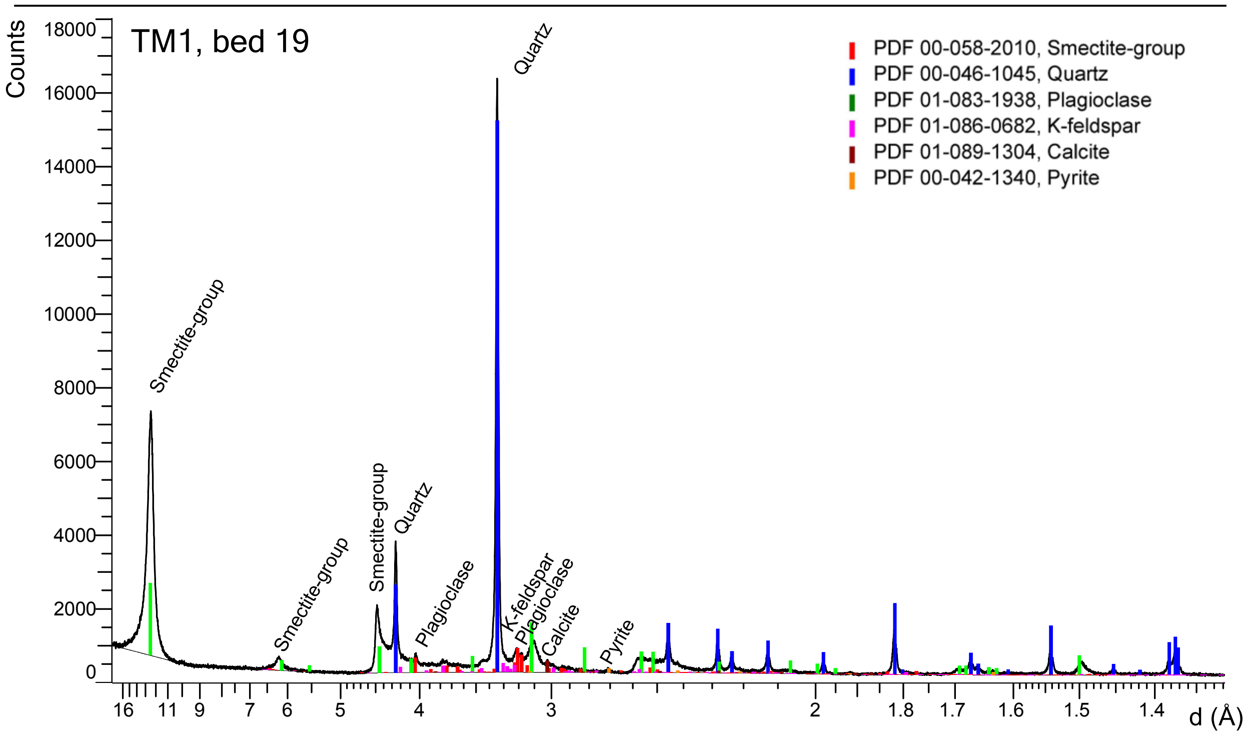

Fig. 1) By XRD intensity, the major compoments of the sample TM1, bed 19 include 12.4 Å smectite-group clay and quartz. K-feldspar, plagioclase, calcite and pyrite are minor components.

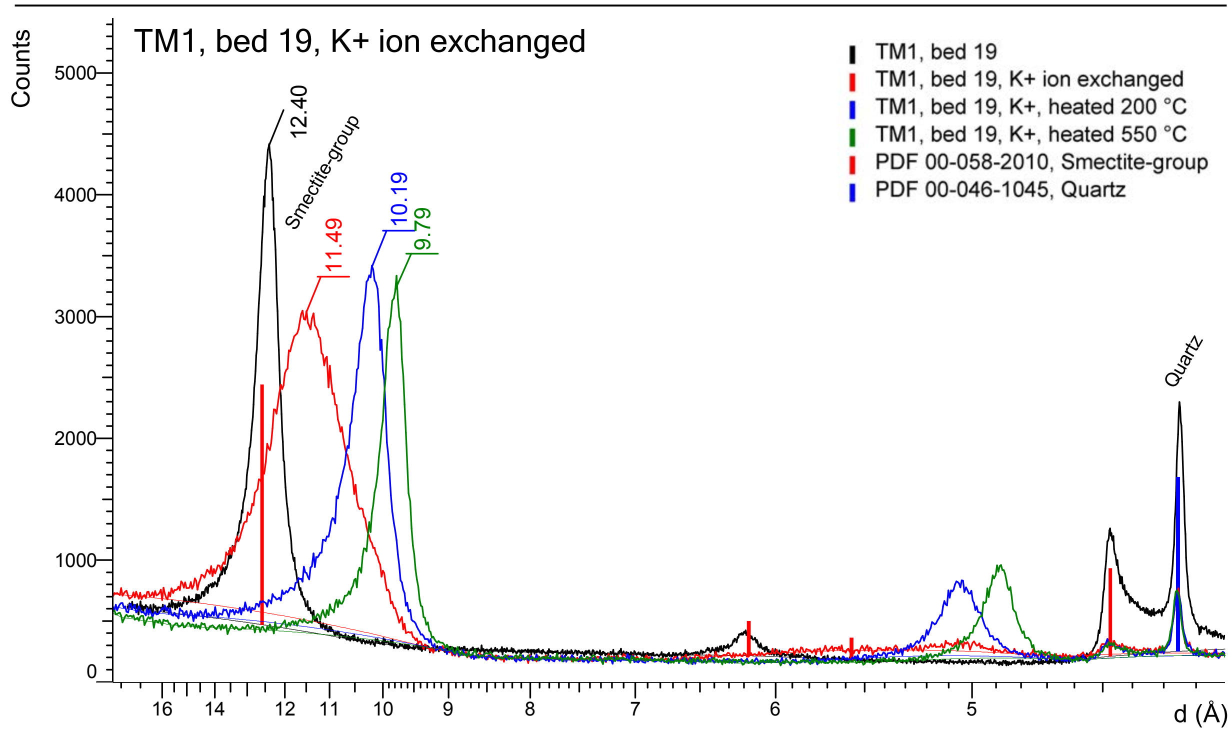

Fig. 2) In the sample TM1, bed 19, the 12.4 Å smectite-group clay mineral peak widens and shifts to 11.5 Å position after K+ ion exchange. Heating to 200°C moves the peak to 10.2 Å and 550 °C to 9.8 Å, which is consistent with smectite-group clay behaviour. No other clay mineral phases appear to be present in the sample.

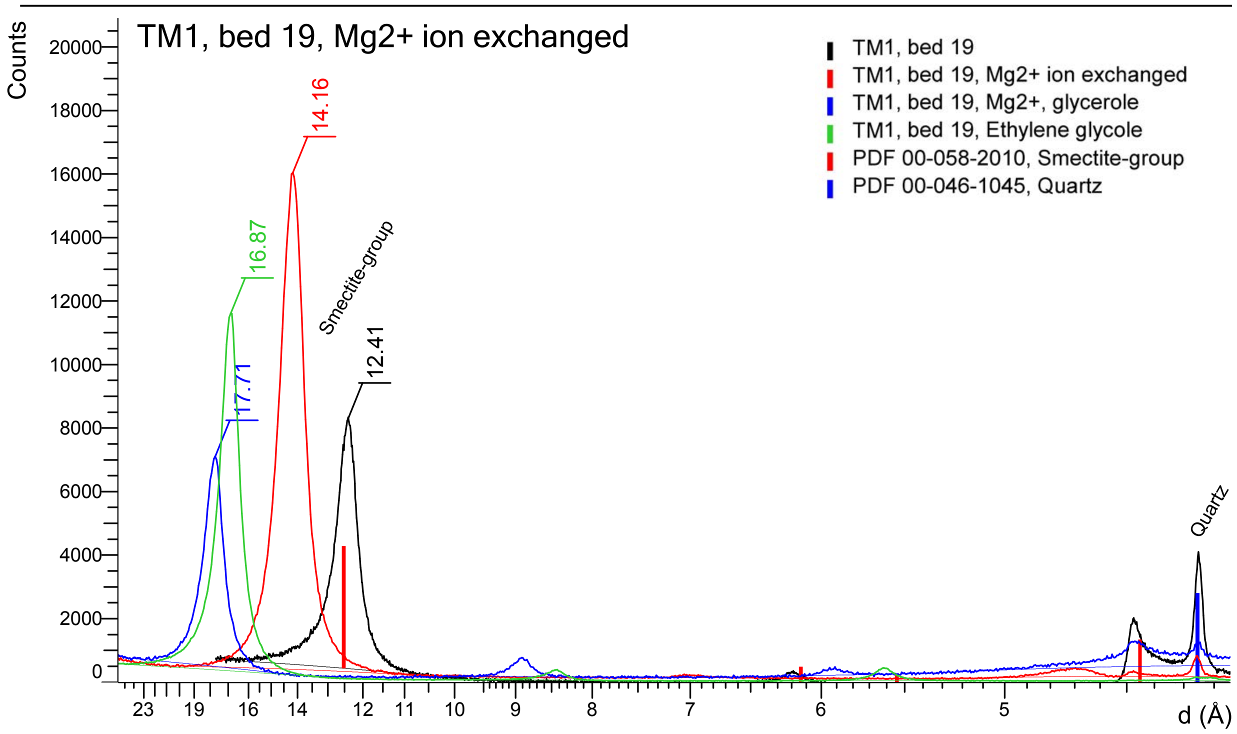

Fig. 3) After Mg2+ ion exchange, the 12.4 Å peak of the smectite-group clay in sample TM1, bed 19 shifts to 14.2 Å position, and glycerole treatment shifts it further to 17.7 Å. Ethylene glycole treatment without the Mg2+ ion exchange results a slightly smaller shift to 16.9 Å.

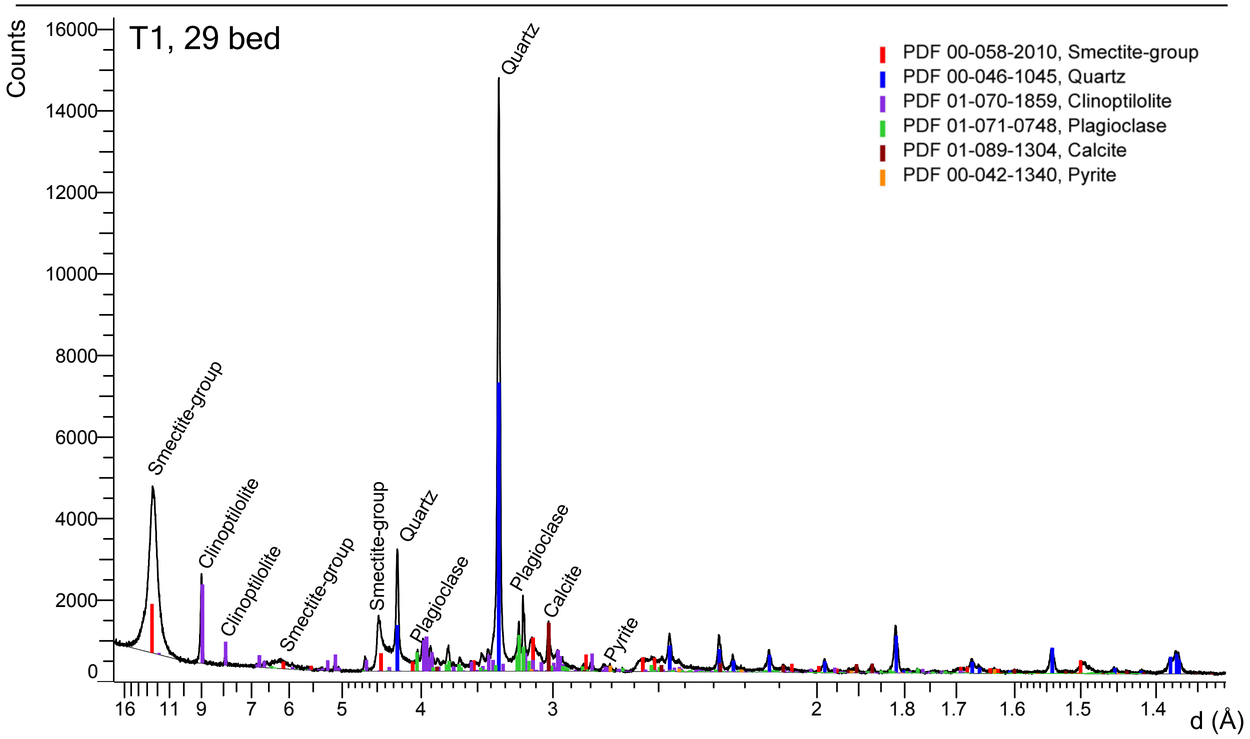

Fig. 4) The main components of the sample T1, 29 bed are quartz and smectite-group clay, but there is also some clinoptilolite, a zeolite-group mineral. Minor components include plagioclase, accessory calcite and pyrite.

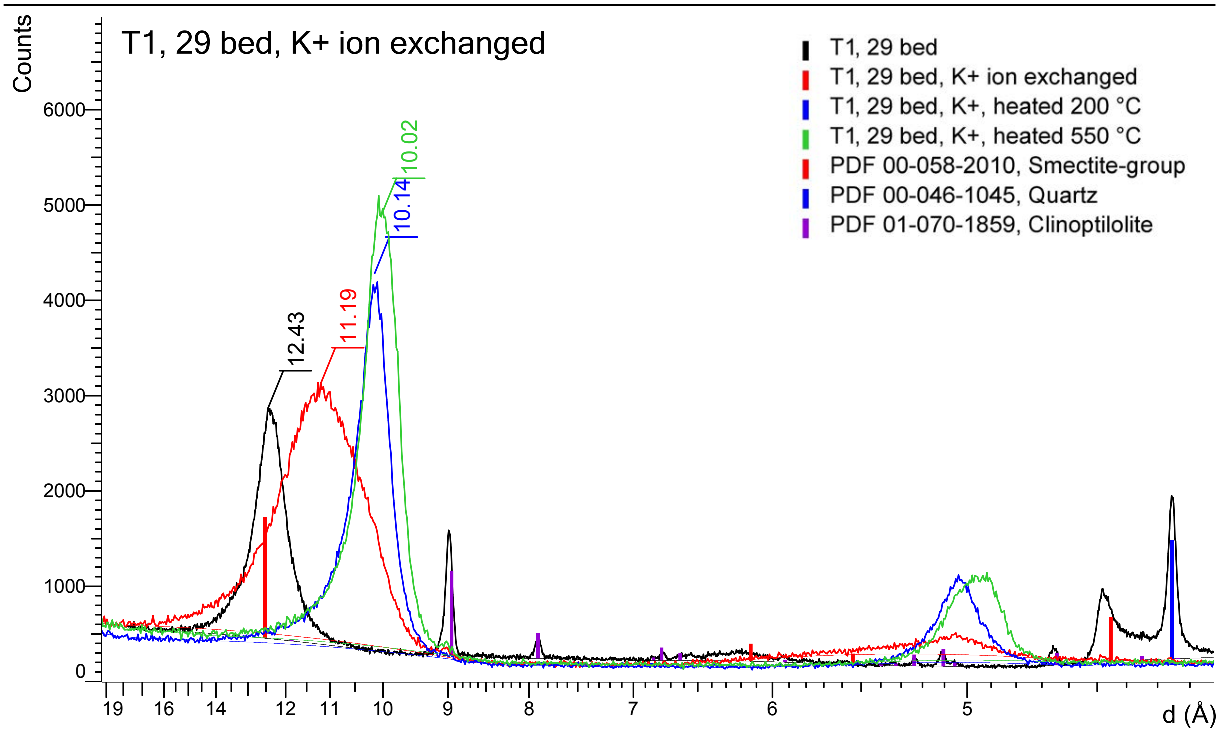

Fig. 5) In sample T1, 29 bed, the 12.4 Å smectite-group clay peak shifts to 11.2 Å position after K+ ion exchange. Heating to 200°C moves it to 10.1 Å and 550 °C to 10.0 Å, as expected for smectites.

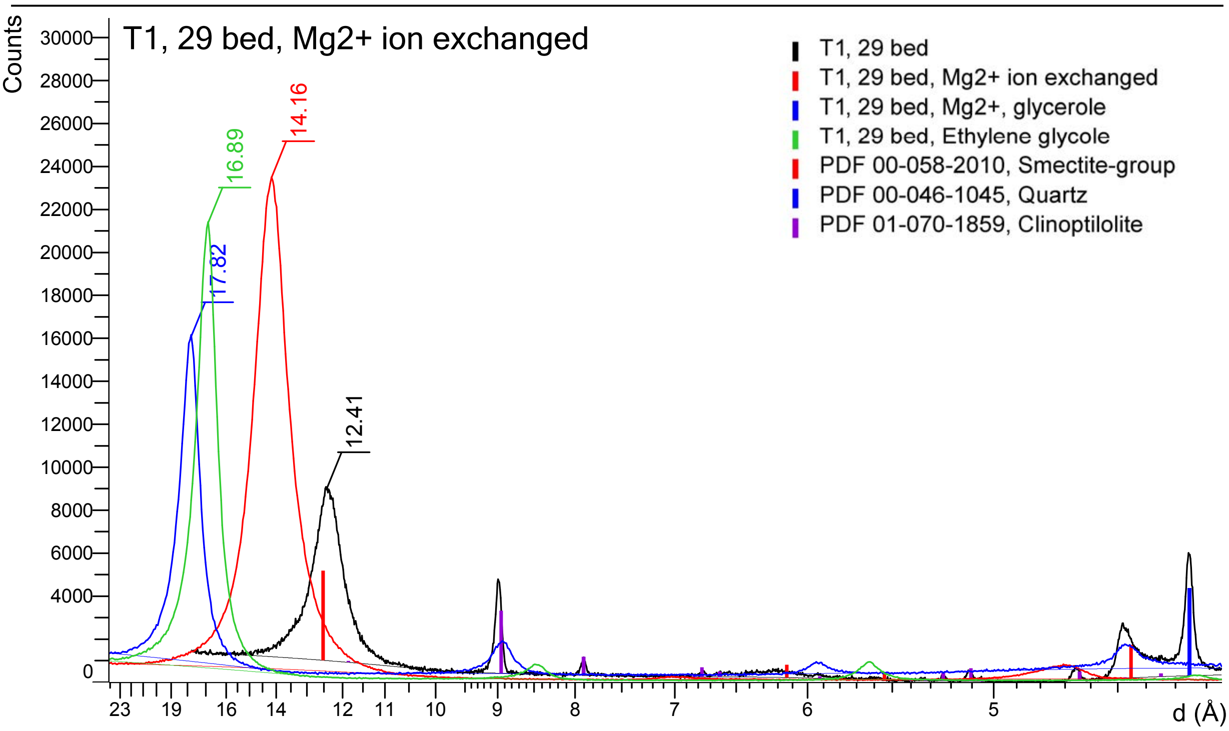

Fig. 6) After Mg2+ ion exchange for the sample T1, bed 29, the 12.4 Å smectite-group clay peak shows swelling to 14.2 Å position, and glycerole treatment shifts it further to 17.8 Å. Ethylene glycole treatment without the Mg2+ ion exchange results a slightly smaller shift to 16.9 Å.

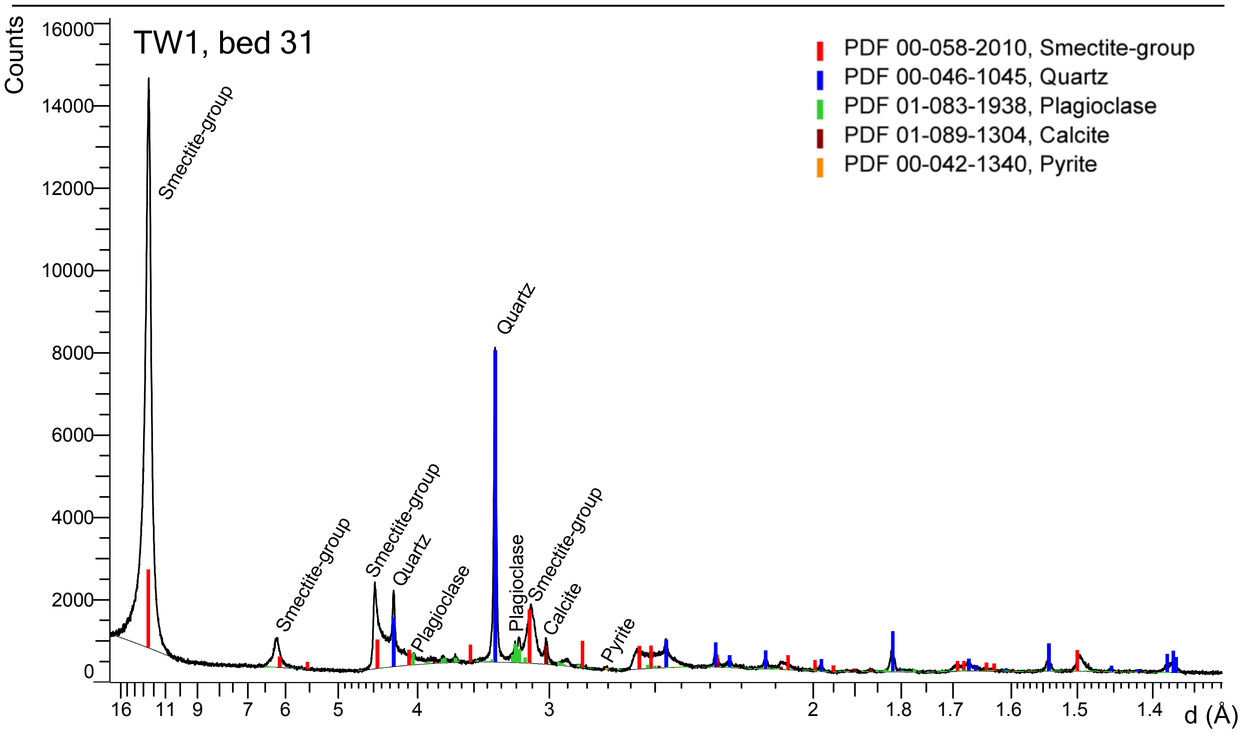

Fig. 7) In the sample TW1, bed 31, the major minerals are smectite-group clay and quartz. Accessory phases include plagioclase, calcite and very small concentration of pyrite.

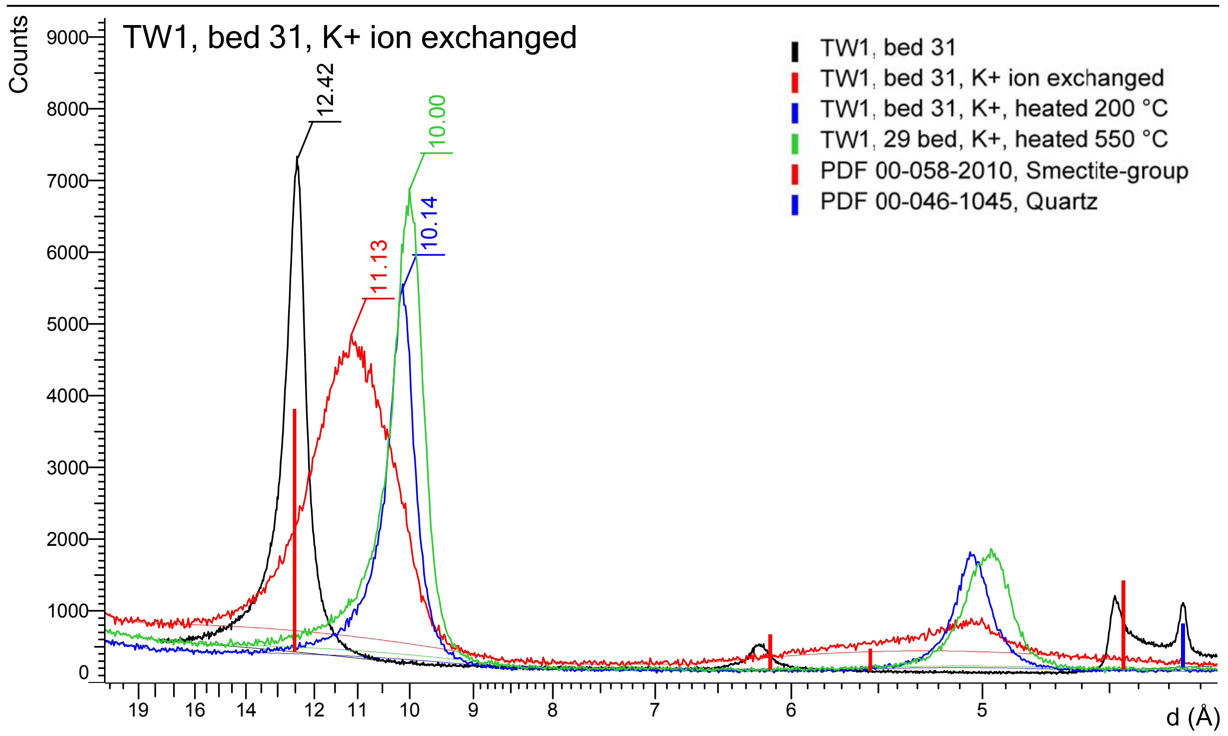

Fig. 8) In sample TW1, bed 31, after the K+ ion exchange, the 12.4 Å smectite-group clay peak widens and shifts to 11.1 Å. Heating for 1 hour in 200°C moves the peak further to 10.1 Å and 550 °C to 10.0 Å, which is consistent with smectite behaviour.

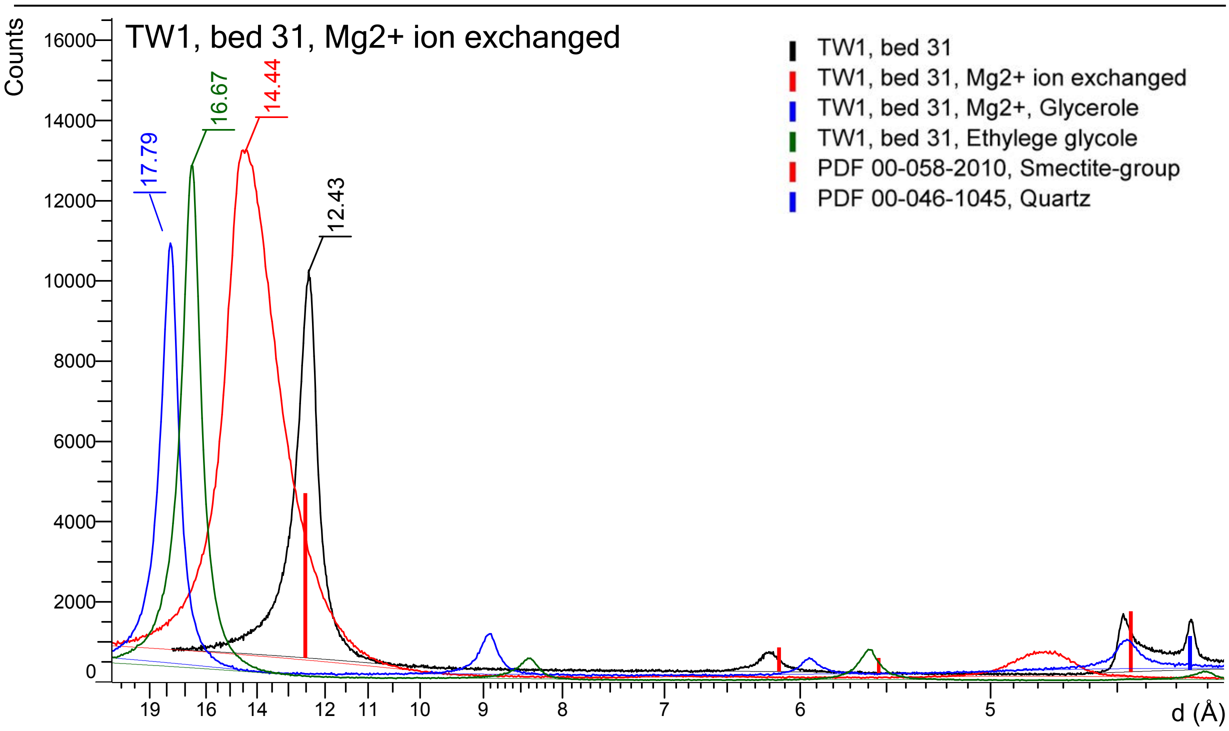

Fig. 9) After Mg2+ ion exchange for the sample TW1, bed 31, the 12.4 Å smectite-group clay peak swells to 14.4 Å, and glycerole treatment shifts it further to 16.7 Å. Ethylene glycole treatment without ion exchange results a shift to 16.7 Å. The swelling behaviour confirms the smectite-group identification and no other clay components are found.

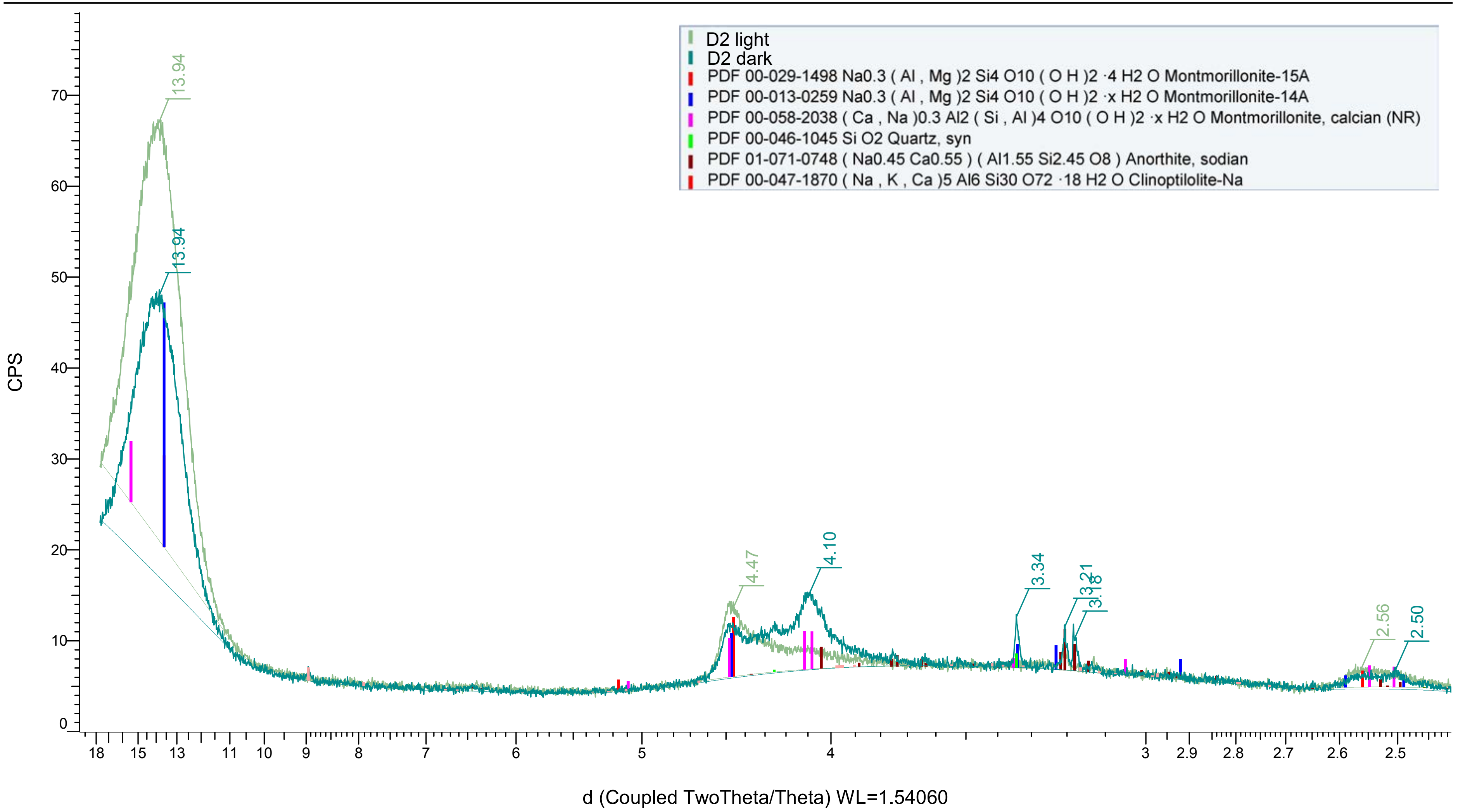

Fig. 10) XRD intensity results showing strong peaks of smectite and some accessory plagioclase (lighter bentonite) and plagioclase, quartz and zeolite (potential clinoptilolite) as accessory mineral.
